# Antibody Fc-chimerism and effector functions: When IgG takes advantage of IgA

**DOI:** 10.3389/fimmu.2023.1037033

**Published:** 2023-02-02

**Authors:** Andréa Cottignies-Calamarte, Daniela Tudor, Morgane Bomsel

**Affiliations:** ^1^ Laboratory of Mucosal Entry of HIV-1 and Mucosal Immunity, Department of Infection, Immunity and Inflammation, Cochin Institute, Paris, France; ^2^ Université Paris Cité, Institut Cochin, Institut National de la Santé et de la Recherche Médicale (INSERM), Centre National de la Recherche Scientifique (CNRS), Paris, France

**Keywords:** antibody-engineering, therapeutic antibodies, Fc-mediated effector functions, chimeric antibody, FcγR, FcαR/CD89, IgA

## Abstract

Recent advances in the development of therapeutic antibodies (Abs) have greatly improved the treatment of otherwise drug-resistant cancers and autoimmune diseases. Antibody activities are mediated by both their Fab and the Fc. However, therapeutic Abs base their protective mechanisms on Fc-mediated effector functions resulting in the activation of innate immune cells by FcRs. Therefore, Fc-bioengineering has been widely used to maximise the efficacy and convenience of therapeutic antibodies. Today, IgG remains the only commercially available therapeutic Abs, at the expense of other isotypes. Indeed, production, sampling, analysis and related *in vivo* studies are easier to perform with IgG than with IgA due to well-developed tools. However, interest in IgA is growing, despite a shorter serum half-life and a more difficult sampling and purification methods than IgG. Indeed, the paradigm that the effector functions of IgG surpass those of IgA has been experimentally challenged. Firstly, IgA has been shown to bind to its Fc receptor (FcR) on effector cells of innate immunity with greater efficiency than IgG, resulting in more robust IgA-mediated effector functions *in vitro* and better survival of treated animals. In addition, the two isotypes have been shown to act synergistically. From these results, new therapeutic formats of Abs are currently emerging, in particular chimeric Abs containing two tandemly expressed Fc, one from IgG (Fcγ) and one from IgA (Fcα). By binding both FcγR and FcαR on effector cells, these new chimeras showed improved effector functions *in vitro* that were translated *in vivo*. Furthermore, these chimeras retain an IgG-like half-life in the blood, which could improve Ab-based therapies, including in AIDS. This review provides the rationale, based on the biology of IgA and IgG, for the development of Fcγ and Fcα chimeras as therapeutic Abs, offering promising opportunities for HIV-1 infected patients. We will first describe the main features of the IgA- and IgG-specific Fc-mediated signalling pathways and their respective functional differences. We will then summarise the very promising results on Fcγ and Fcα containing chimeras in cancer treatment. Finally, we will discuss the impact of Fcα-Fcγ chimerism in prevention/treatment strategies against infectious diseases such as HIV-1.

## Highlights

- Antibody-based therapies take advantage of the Fc region of antibodies to activate a broad spectrum of Fc-mediated effector functions, thereby linking adaptive and innate immunity.
- IgA conveys stronger FcR signalling and effector functions than IgG, but evaluation of IgA therapeutics is neglected.
- The presence of both Fcα and Fcγ gives the resulting chimeric antibodies the ability to bind to a greater number of FcR molecules than the parental antibodies, resulting in increased killing of target cancer cells.
- Chimeric Abs containing Fcα-Fcγ could be effective as anti-infective agents, particularly against HIV-1.

## 1 Introduction

Antibodies (Abs) used as therapeutic agents have many advantages such as specific targeting provided by their Antigen Binding Fragment (Fab), good bioavailability, and the ability to engage immune effector cells using their crystallizable region (Fc). In humans, IgG and IgA predominate over the other three Ab isotypes. IgG accounts for more than 80% of circulating Abs (IgG1 being the most abundant (1)), and with a proportion of 15%, IgA (mainly IgA1) is the second most abundant circulating isotype (2). In contrast, in the mucosa, more than 90% of mucosal antibodies are IgA, with the ratio of IgA1 to IgA2 subtypes varying according to mucosal site (2).

**Figure 1:**
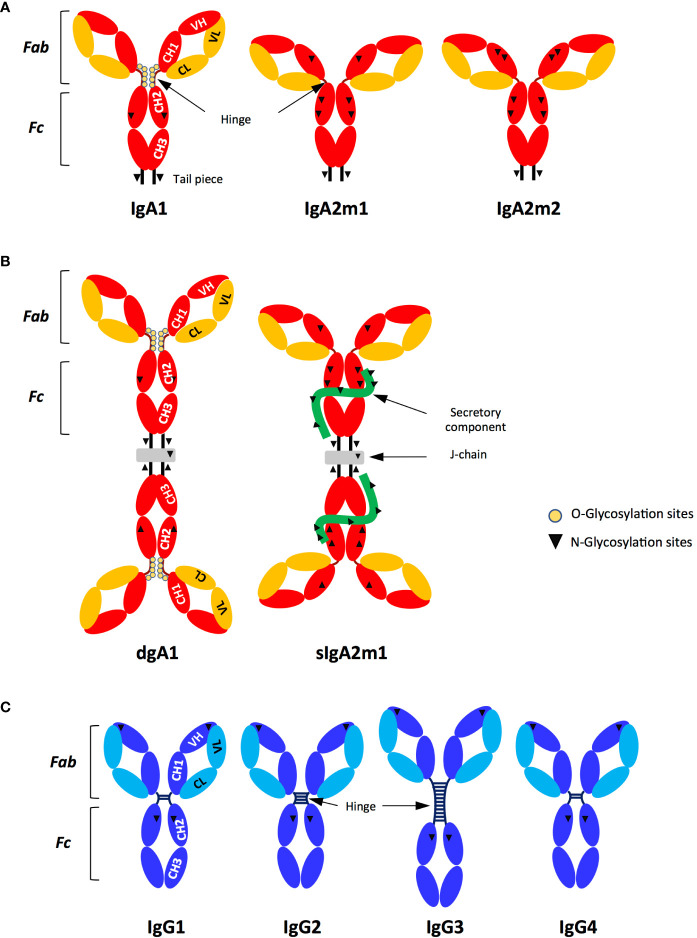
Structure and subtypes of IgG and IgA. **(A)** Monomeric IgA (IgA1, A2m1, IgA2m2); **(B)** Dimeric IgA (dIgA1) and secretory IgA (sIgA2m1). **(C)** IgG (IgG1, IgG2, IgG3, IgG4). The IgA **(A, B)** and IgG **(C)** isotypes contain two functional parts: the Fab formed by the entire light chain (VL-CL) and the first two domains of the heavy chain (VH-CH1) while the Fc is formed by the CH2 and CH3 domains located in the constant heavy chains. The VH and VL sequences represent the variable regions of the heavy and light chains (HC and LC respectively), together forming the antigen-specific paratope. The J chain (grey) is present in dimeric and secretory IgA (dIgA and sIgA, respectively). SIgA contains the secretory component (green), which corresponds to the extracellular region of pIgR acquired by proteolytic cleavage, after pIgR had translocated dimeric IgA to mucosal surfaces **(B)**. Inverted triangles and yellow circles represent the N- and O-glycosylation sites, respectively (A, B and C). Hinge length varies between human IgA and IgG subtypes: **(A, B)** IgA1>IgA2; **(C)** IgG3>IgG2>IgG1 and IgG4.

IgA and IgG share common features, including general shape and functional domains (**Figure 1**). Both isotypes bind to the antigen using their Fab, which is linked *via* a hinge region to the Fc. The latter then interacts with the corresponding Fc receptor (FcR) on the surface of the effector cell. However, IgG and IgA differ in their glycosylation patterns, with IgG showing predominantly N-glycosylations while IgA shows N- and O-glycosylations, even in the hinge region (3) (**Figures 1A, C** **)**. In addition, a tail completes the C-terminus of the IgA heavy chain (HC), which contributes to the dimerisation/multimerisation processes of mucosal IgA through binding to the J-chain (2) (**Figure 1B**). Despite the compartmentalisation of Ab isotypes that has evolved to counteract tissue-specific pathologies, the current development of therapeutic Abs focuses primarily on IgG, particularly IgG1. This predominance is the result of various technical factors: (i) IgG is the most abundant isotype in blood, easier to produce in large quantities and, therefore, widely studied, unlike the other isotypes; (ii) conversely, IgA, the second most abundant isotype in serum and the most abundant in mucosal sites, is more difficult to sample, produce and purify; (iii) IgG1 and IgG3 are the IgG subtypes that best induce Fc-dependent effector functions; (iv) IgG1 has a longer half-life in blood than IgG3 and IgA; and (v) IgG3, due to a long hinge, like IgA1 (**Figures 1A, C**), and a complex glycosylation profile, is produced at a higher cost than IgG1 (4–7). Thus, IgG1 has become the preferred isotype for the design of therapeutic antibodies. This choice should be reconsidered because of isotypic differences in Fc-mediated functions that are particularly significant for IgA.

To determine how antibody functions can be improved to meet therapeutic needs, a thorough understanding of antibody biology is required. Therefore, a variety of Fc-mediated functions of IgG and IgA have been extensively characterised over the past 20 years, revealing substantial IgA efficiencies that could be applied to the design of therapeutic mAbs (8). Abs link adaptive and innate immunity by interacting with their corresponding FcR on the surface of effector cells downstream of antigen targeting, resulting in the induction of a wide range of immune responses (8–10). In cancer treatment, one of the main therapeutic Ab mechanisms is initiated by opsonisation of cancer cells, leading to their elimination by innate immune effector cells (11). However, despite the superior ability of IgA-FcαRI/CD89 compared to IgG/FcγR to trigger effector functions such as antibody-dependent cellular cytotoxicity (ADCC) or antibody-dependent cellular phagocytosis (ADCP), the use of IgA as a therapeutic agent remains limited (12–16). To take advantage of the signalling cascades and subsequent effector functions mediated by both isotypes, chimeras containing two Fc domains, namely Fcγ and Fcα in tandem, have emerged as new formats of therapeutic Abs (17, 18). This approach is also relevant for mucosal pathogens, such as Human Immunodeficiency Virus type 1 (HIV-1). Thus, prevention of viral entry into mucosa by HIV-1-specific IgA (19, 20) as well as neutralisation of HIV-1 in the circulation by HIV-1-specific IgG correlate with protection against HIV-1 infection *in vivo* (19–22).

In this review, we detail the differences between IgG- and IgA-dependent intracellular FcR signalling pathways and Fc-mediated effector functions, and describe how new Ab-based therapeutic strategies can benefit from these properties, especially in the field of Aqcuiered Immunodeficiency Syndrome (AIDS).

## 2 The basis of FcR-mediated functions

The Fc adopts two dominant conformational states, namely an open and relaxed ‘U’ shape or a closed and compact ‘V’ shape, mainly regulated by N-glycosylation. Each conformation of Fcγ engages a distinct class of FcRs, namely type I and type II FcRs, and in turn activates various effector functions. The open ‘U’ form binds preferentially to type I FcRs while the closed ‘V’ form binds preferentially to type II FcRs (1, 9, 23). There are also other FcRs for IgG, such as TRIM21 and the neonatal FcR (FcRn), for which no preferential conformation of Fcγ is required for binding (9, 24, 25). In contrast, the role of Fcα conformation in IgA binding of FcRs has been less described (8, 26–29), although FcαRI/CD89, the major FcαR, appears to be a type I FcR.

An additional parameter that affects Fc-mediated functions is the oligomerisation state of Ab. Compared to IgG, which is exclusively expressed as a monomeric antibody, IgA is predominantly monomeric in serum and polymeric (mostly dimeric) in mucosal tissues. Dimeric IgA becomes secretory (sIgA) when it is secreted to mucosal surfaces after acquisition by proteolytic cleavage of the secretory component (SC), the extracellular part of the polymeric Ig receptor (pIgR) which transcytoses IgA and allows it access to external fluids (8). Furthermore, the SC protects IgA from degradation (**Figures 1A, B** **)**.

### 2.1 Understanding the Ab-FcR interaction

**Figure 2:**
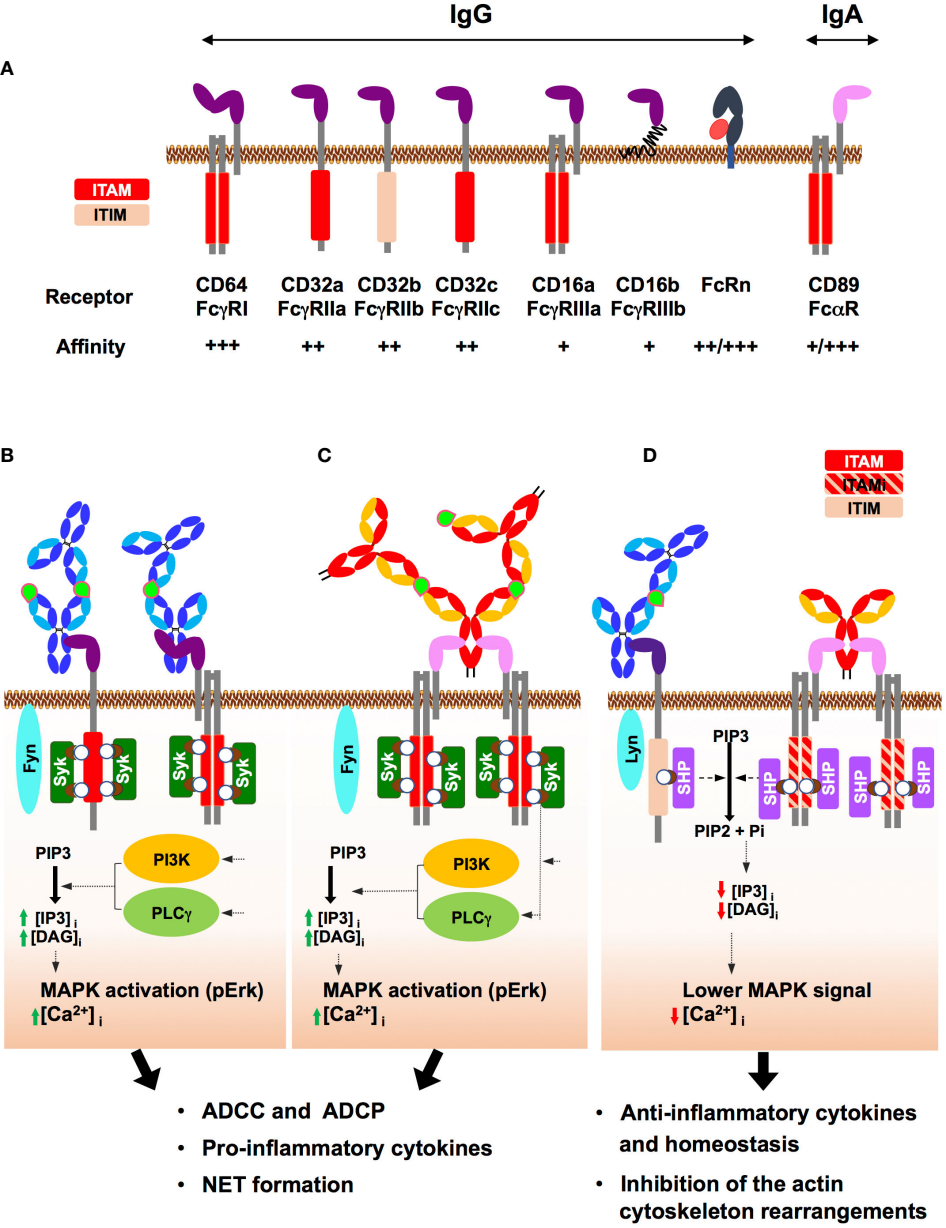
Human IgG and IgA Fc receptors (FcRs) and their mode of action. **(A)** Major IgG and IgA FcRs: IgG and IgA FcRs are represented at the cell membrane, associated where indicated with the adaptor protein FcRγ. The respective affinities for IgG from + to +++ are indicated. For FcRn and FcαRI/CD89, ++/+++ and +/+++ refer to the affinity level of antigen-free/antigen-bound antibodies. The activation (ITAM) and inhibition (ITIM) motifs of tyrosine-based immunoreceptors are shown in red and light pink, respectively. The neonatal Fc receptor (FcRn), a non-classical MHC class I only capable of interacting with IgG, is shown associated with its β2-microtubulin chain (red). **(B)** ITAM-induced activation upon FcγR binding by IgG-IC: IgG binding in the immune complex (IgG-IC) (antigen in light green) to FcγRI/CD64 or FcγRIIa/CD32a results in FcR clustering that triggers the Src kinase Fyn. In turn, Fyn phosphorylates the two tyrosines (white circle) of ITAM contained either in the cytosolic tail of the FcR or in the associated adaptor protein (FcRγ chain). These phosphorylations generate docking sites for Syk kinase family that induces a PI3K- and PLCγ-mediated intracellular increase in IP3 and DAG, followed by MAPK activation and an increase in intracellular Ca2+. Subsequently, effector functions such as ADCC and ADCP as well as cytokine secretion and NETosis are triggered. **(C)** ITAM-induced activation upon FcαRI/CD89 binding by IgA-IC: IgA-IC cross-links FcαRI/CD89 and the associated FcRγ chain, initiating ITAM phosphorylation by Fyn and the subsequent signalling pathway, similar to that triggered above for IgG-IC. In turn, effector immune cells are activated as well as a pro-inflammatory response, resulting in pathogen elimination by ADCC and ADCP but also cytokine secretion, and NETosis. **(D)** ITIM/ITAMi signalling: Binding of IgG-IC to FcγRIIb/CD32b carrying ITIM induces monophosphorylation of ITIM. Binding of uncomplexed IgA to FcαRI/CD89 triggers suboptimal phosphorylation of ITAM on the intracellular adaptor FcRγ, termed ITAM inhibitor (ITAMi) and schematised in a red/pink hatched rectangle. Then, the ITAMi and ITIM pathways converged: Monophosphorylation of ITIM or ITAMi by Lyn, another receptor-associated Src kinase, promotes the recruitment of SH2-phosphatases (SHP). In turn, SHP opposes Syk activity, thereby aborting the immune response. These two different negative regulatory mechanisms, ITIM and ITAMi, help prevent uncontrolled inflammation and maintain immune homeostasis.

IgG can bind to six different type I IgG-specific FcRs, namely FcγRI/CD64, FcγRII/CD32a, b and c, and FcγRIII/CD16a and b (**Figure 2A**). These type I FcRs are either pro-inflammatory such as FcγRI/CD64, FcγRIIa/CD32a, FcγRIIc/CD32c, FcγRIIIa/CD16a and FcγRIIIb/CD16b, or anti-inflammatory such as FcγRIIb/CD32b (9). The balance between inflammatory and anti-inflammatory properties relies on induced cell signalling, and the subsequent release of specific sets of cytokines, as discussed below. IgG interacts with the type I FcR *via* the upper part of the CH2 domain of Ab located next to the hinge region that activates downstream signals *via* an immunoreceptor tyrosine-based activation or inhibition motif (ITAM and ITIM, respectively) (30). ITAM is contained either in the cytoplasmic domain of the receptor namely the FcγRIIa/CD32a and FcγRIIc/CD32c, or in the γ-chain, the FcR common adaptor associated with the FcγRI/CD64 and FcγRIIIa/CD16a, resulting in a cascade of activation signals (**Figure 2A**). Activation of ITAM engages a pro-inflammatory signalling pathway in effector cells while the ITIM domain activates a downstream anti-inflammatory signalling cascade, described below. FcγRIIb/CD32b is the only inhibitory FcγR that carries the ITIM motif in its cytoplasmic domain (**Figure 2A**), thus controlling many aspects of the inflammatory response. Among IgG isotypes, IgG3 has the highest affinity for FcγRI/CD64, followed by IgG1 and IgG4, while IgG2 is unable to bind to FcγRI/CD64 (10).

IgA binds to two different type I FcRs: FcαRI/CD89 and Fcα/µRI. IgA binds to FcαRI/CD89 *via* the CH2 and CH3 domains and, like FcγRI/CD64, FcαRI/CD89 associates with the FcRγ adapter chain to induce downstream signalling (8) (**Figure 2A**). The affinity of FcαRI/CD89 for IgA varies according to its ability to form an immune complex (IC) with the antigen, whether free or on the surface of opsonised cells or microbes. The affinity of FcαRI/CD89 for IgA is low when IgA is free of antigen but becomes high when IgA is in IC with its antigen. As will be discussed below, FcαRI/CD89 can propagate activating but also inhibitory signalling pathways, called inhibitory ITAMs (ITAMi), through the same ITAM domain of the FcRγ chain when activated by antigen-free IgA. Monomeric and dimeric IgA engage FcαRI/CD89 in an IgA:receptor molecular ratio of 1:2, but the engagement of secretory IgA is limited by the presence of the SC that partially masks the FcαRI/CD89 interaction domain (27, 31–33). Fcα/µRI is much less well characterized than FcαRI/CD89. It is expressed by marginal zone B cells, follicular DCs and tonsil cells with expected functional roles (34), but not by circulating lymphocytes.

Type II FcRs are C-type lectins, such as DC-SIGN or Dectin-1, which bind to carbohydrate biomolecules, such as Abs. In contrast to type I FcRs, type II FcRs preferentially interact with the closed ‘V’ conformation of the Fc domains of IgG but also of IgA. Ab binding triggers anti-inflammatory responses (35, 36), although these results remain debated (37–39).

As summarised in **Table 1**, FcRs are predominantly expressed on innate immune cells which are therefore considered to be the main effector cells. The expression of FcRs on immune cells throughout the human body is well documented, but comparative expression of FcγR and FcαR between blood cells and mucosal tissues is lacking (40–42). FcR expression varies qualitatively and quantitatively between blood DC subsets (43), but also between blood and tissue DCs and different mucosal DCs. Genital and rectal mucosal DCs express FcγRII/CD32, low levels of FcαRI/CD89 and FcγRI/CD64 but lack FcγRIII/CD16. Blood DCs have a similar pattern of FcR expression to tissue DCs, although the level of FcγRII/CD32 (a, b and c) is higher and that of FcγRIII/CD16 is just detectable in blood DCs (40). Blood and mucosal NK cells express only FcγRIII/CD16 but not FcαRI/CD89 in men and at low frequency in the female genital tract correlating with a sexual polymorphism (40).

**Table 1:** Expression pattern of major FcR for IgG and IgA in human immune and non-immune cells.

	Cell type	FcγRI	FcγRIIa	FcγRIIb	FcγRIIc	FcγRIIIa	FcγRIIIb	FcαRI	DC-SIGN	pIgR	FcRn	TRIM21
Blood	B lymphocytes											
	T Lymphocytes											
	NK cells											
	Monocytes											
	Dendritic cells											
	Neutrophils											
	Eosinophils											
	Basophils											
	Mast cells											
Mucosa	Mucosal Epithelium											
	B lymphocytes											
	T Lymphocytes											
	NK cells											
	Monocytes											
	Dendritic cells											
	Macrophages											
	Neutrophils											
	Eosinophils											
	Basophils											
	Mast cells											

Expression level: None 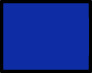 Very Low 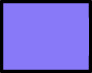 Low 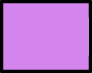 Intermediate 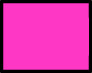 High 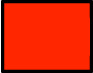

All IgA and IgG FcRs are expressed by mucosal macrophages in genital and rectal tissues, whereas in the gastrointestinal tract, macrophages are almost devoid of FcRs and express only very low levels of FcγRI/CD64 and FcγRII/CD32. In contrast, blood monocytes all express FcRs at a high level (40, 41, 44). No comparison of FcR expression between blood and mucosal neutrophils has been reported, although cervical and colonic neutrophils express FcγRIII/CD16 and FcαRI/CD89 in a similar manner (45). Furthermore, we found that blood neutrophils express only FcγRII/CD32 and FcαRI/CD89 (46).

### 2.2 Canonical type I FcR signalling

The high-affinity type I receptors, FcγRI/CD64 and FcαRI/CD89, both use the same intracellular machinery *via* ITAM signalling motifs found in the cytoplasmic tail of the FcRγ chain, albeit the stoichiometry of IgA and IgG binding to their cognate FcR is different. Indeed, while only one Fcγ of the IgG binds to FcγRI/CD64 in a IgG:FcγRI/CD64 molar ratio of 1:1, each Fcα of the IgA recruits one FcαRI/CD89 in a IgA:FcαRI/CD89 molar ratio of 1:2 (8). Therefore, for an equal amount of Abs, the signalling cascade induced by IgA-IC *via* FcαRI/CD89 is twice that induced by IgG-IC *via* FcγRI/CD64 (13). Worthnoting, monomeric and dimeric IgA have comparable affinities for FcαRI/CD89, suggesting that FcαRI/CD89 binds to both forms of IgA in a similar 2:1 stoichiometry (26, 33, 47). Similar to the interaction of FcγRI/CD64 with IgG, the medium and low affinity IgG receptors FcγRII/CD32 and FcγRIII/CD16 bind to the Fcγ region in a 1:1 molar ratio (10).

These Ab : FcR interactions lead to three types of intracellular signalling cascades as follows.

The first two types are triggered only by Abs in IC with free antigen or when they coat the surface of opsonised cells or microbes that can cross-link the receptors and in turn trigger cell activation (**Figures 2B, C** **)**. During IgG-IC-induced activation, the Src Fyn tyrosine kinase phosphorylates at several sites either the FcR ITAM, i.e. FcγRIIa/CD32a, or the intracellular FcRγ-chain adaptor associated with FcγRI/CD64 (**Figure 2B**). Upon IgA-IC-induced activation, the Src Fyn tyrosine kinase phosphorylates the intracellular FcRγ-chain adaptor associated with FcαRI/CD89 (**Figure 2C**). In both cases, this phosphorylation allows the recruitment of SH2-adaptor proteins Syk, which in turn activate PI3K and PKC kinases, ultimately triggering the ERK pathway in association with an increase in cytoplasmic Ca2+ concentration. Together, these actors are responsible for cell activation, phagocytosis, ROS and NET formation, cytokine release and antigen presentation.

In contrast, the last type of intracellular signalling cascades is triggered when IgG forms an IC and engages FcγRIIb/CD32b but also when IgA is devoid of antigen. These two cases of Ab : FcR interactions lead to regulatory inhibition that contributes to immune homeostasis (**Figure 2D**). In fact, IgG-IC binds to FcγRIIb/CD32b containing ITIMs that become monophosphorylated. Alternatively, antigen-free IgA binds to FcαRI/CD89 triggering suboptimal phosphorylation of the ITAM-containing intracellular adaptor FcRγ, which is called ITAMi. In turn, monophosphorylation of ITIM and ITAMi recruits SH2-phosphatases that counteract Syk activity (48). Therefore, activations of ITIM and ITAMi compensate that of ITAM signalling in the same cells (30, 48). Suboptimal stimulation of FcγRIIa/CD32a by anti-FcγRIIa/CD32a Fab has also been reported to trigger ITAMi signalling in mice (9, 13, 30, 42, 49, 50). However, no suboptimal phosphorylation of ITAM (i.e. ITAMi signalling) has been reported upon engagement of uncomplexed IgG with FcγRI/CD64 and FcγRIIIb/CD16b, the latter associated with the ITAM-bearing adaptor chain FcRγ, probably because the affinity of antigen-free IgG for both receptors is too low.

Although IgA can mediate signalling when it is devoid of antigen, IgG acts in the context of immune complexes *in vivo*. In this later case, the size of the immune complex, as well as Ab subclass composition and glycosylation contribute to the strength of FcR engagement and downstream effector functions (51). At the cellular level, the effector cell typically expresses multiple FcRs that can each be engaged by the multiple Fc domains of the Abs comprised in an IC. Therefore, activation (ITAM) and inhibition (ITIM and ITAMi) signals can be triggered simultaneously on effector cells. The resulting net effector function will depend on the balance between the extent of anti-inflammatory and pro-inflammatory FcRs signalling induced.

### 2.3 Other non-canonical FcRs

Two non-canonical FcRs have been described. The first, TRIM21, has been proposed to act as an intracellular cytoplasmic FcR for IgG and IgA (monomers or dimers) (52–54). By binding to Abs, TRIM21 triggers intracellular degradation of virus-Ab immune complexes by the Ub-ligase activity of TRIM21. This mechanism is independent of Ab neutralising activity, although it is referred to as Antibody-Dependent Intracellular Neutralization (ADIN). Indeed, ADIN refers to the neutralisation of the virus due to its degradation rather than the ability of Ab to directly block virus entry and infection of target cells (52–54). In addition, TRIM21 facilitates antiviral functions through proteasomal degradation, and exposure of the viral genome to cellular sensors (25, 52, 55).

The second, FcRn, a non-classical MHC-I molecule, is ubiquitously expressed. FcRn binds Ab only at low pH, which occurs after endocytosis of Ab by different FcγRs and its release into an acidic compartment. FcRn is responsible for the recycling of antigen-free IgG to the cell surface and its release at neutral pH. This recycling prevents antigen-free IgG from undergoing proteasomal degradation and results in a prolonged systemic half-life of IgG. In contrast, FcRn routes IgG-IC to degradation, resulting in cross-presentation (24). Therefore, binding of IgG to FcRn increases the cross-presentation of peptides to the corresponding specific CD8+ T cells *in vitro* (56), providing an additional bridge between adaptive and innate immunity. FcRn-mediated cross-presentation may have contributed to a superior HIV-1-specific CD8+ T-cell response in non-human primates (NHPs) infected with recombinant simian virus-HIV-1 (SHIV) pre-treated with HIV-1-specific IgG carrying an LS mutation that increases Ab binding to FcRn, compared to untreated animals (57). The role of FcRn in cross-presentation has been further confirmed in animal models of cancer (56, 58–60). This non-classical MHC-I molecule deserves further study. FcRn is also responsible for the bidirectional transport of IgG across the epithelium (24).

Abs can also bind FcR-Like receptors (FCRL), a family of receptors named by homology to the canonical FcγRI/CD64 (61). The FCRL family currently contains 6 members (1–6) and are preferentially expressed on B cells, at least for FCRL1-5. Yet, FCRL3 is also expressed by Th17 and Treg (6a3), while FCRL6 is mainly expressed by certain subsets of T cells and NK cells (62, 63). As recently reviewed (64), the intracellular region of FCRLs contains either a single ITAM domain, as in FCRL1, or an ITAM domain with a variable number of ITIM domains, namely from one to three in FCRL2-6. This heterogeneity in the ITIM/ITAM configuration probably leads to major functional differences between the various FCRL members. Nevertheless, only FCRL1 functions as an activating receptor, the others being considered as negative regulators.

Importantly, only FCRL3, FCRL4 and FCRL5 act as Ab receptors. FCRL3 appears to bind IgA but only in its secretory sIgA form, and interaction of artificially cross-linked sIgA with FCRL3 results in inhibition of Treg functions and promotion of a Th17 phenotype (65). This suggests that FRCL3 may act as a mucosal sensor of ICs promoting a local inflammatory environment, and driving regulatory T cell plasticity to help control pathogen invasion. This hypothesis has yet to be validated using sIgA naturally cross-linked by their specific antigen. In contrast, FCRL4 on memory B cells binds only to serum IgA but not to sIgA (66), and is thought to participate in mucosal tolerance. FCRL4 also binds weakly to heat-aggregated IgG3 and IgG4 (67), although further experiments with native IgG alone or in IC are needed to functionally validate these interactions. FCRL5 binds to all IgG isotypes although with different affinities (67, 68). In FCRL5 transfected cell lines, IgG binding to FCRL5 increases with Ab de-glycosylation, this later increasing with IgG lifetime (68). This result needs to be validated in primary FCRL5+ cells.

The biology and functions of FCRL are poorly understood. It has been proposed that FCRL3, FCRL4 and FCRL5 expressions are involved in B cell development in different B cell subsets (69–71). For example, FCRL5 cross-linking on B cells counteracts BCR signalling while increasing the proliferation index, a major feature for B cell development (69). As recently summarised, FCRL3-5 may exert important roles in mucosal protection, not only as immune-promoting sensors but also as depletion markers in autoimmune disorders and chronic viral infections (72). Overall, cross-linking of FCRLs by IC leading to the elimination of the Ab-targeted cell/microorganism occurs only in rare cases.

### 2.4 *In vivo* models to study the effector functions of antibodies

Animals such as mice or Non-Human Primates (NHPs) are key models to fully understand how Abs mediate effector functions and contribute to pathophysiology. However, one of the key questions in these studies is the specific engagement of Abs with FcRs, whether in mice or NHPs. The affinity of human antibodies for animal FcRs and, conversely, that of animal antibodies for human FcRs differ from an autologous situation. In particular, human IgG has a slightly lower affinity for mouse FcRs (including FcRn) than for human FcRs, and the level of FcγR expression varies between animal and human effector cells. These characteristics may limit the extrapolation of Ab-FcR interaction results from animals to humans (8, 10, 42, 73–76). Furthermore, as mice express pIgR but not FcαRI/CD89, the use of CD89 Tg mice is mandatory when studying IgA effector functions in mouse models (2, 73, 76). Alternatively, NHPs, a more human-like animal model, express all FcγRs, FcαRI/CD89 and pIgR, and are therefore more suitable for preclinical studies on therapeutic Abs, including IgA (73, 74, 77). Some FcγR expression patterns are peculiar, notably in neutrophils. In human, neutrophils express only FcγRIIa/CD32a and FcγRIIIb/CD16b whereas in NHPs, although lacking FcγRIIIb/CD16b, neutrophils express FcγRI/CD64, FcγRIIa/CD32a and FcγRIIb/CD32b. However, Ab and FcR species matching studies in the animal model would require the development of expensive humanised animal models, especially if the various human FcRs are expressed in each lineage and mediate the corresponding effector functions.

### 2.5 Fc-dependent and Ab-mediated effector functions

**Figure 3:**
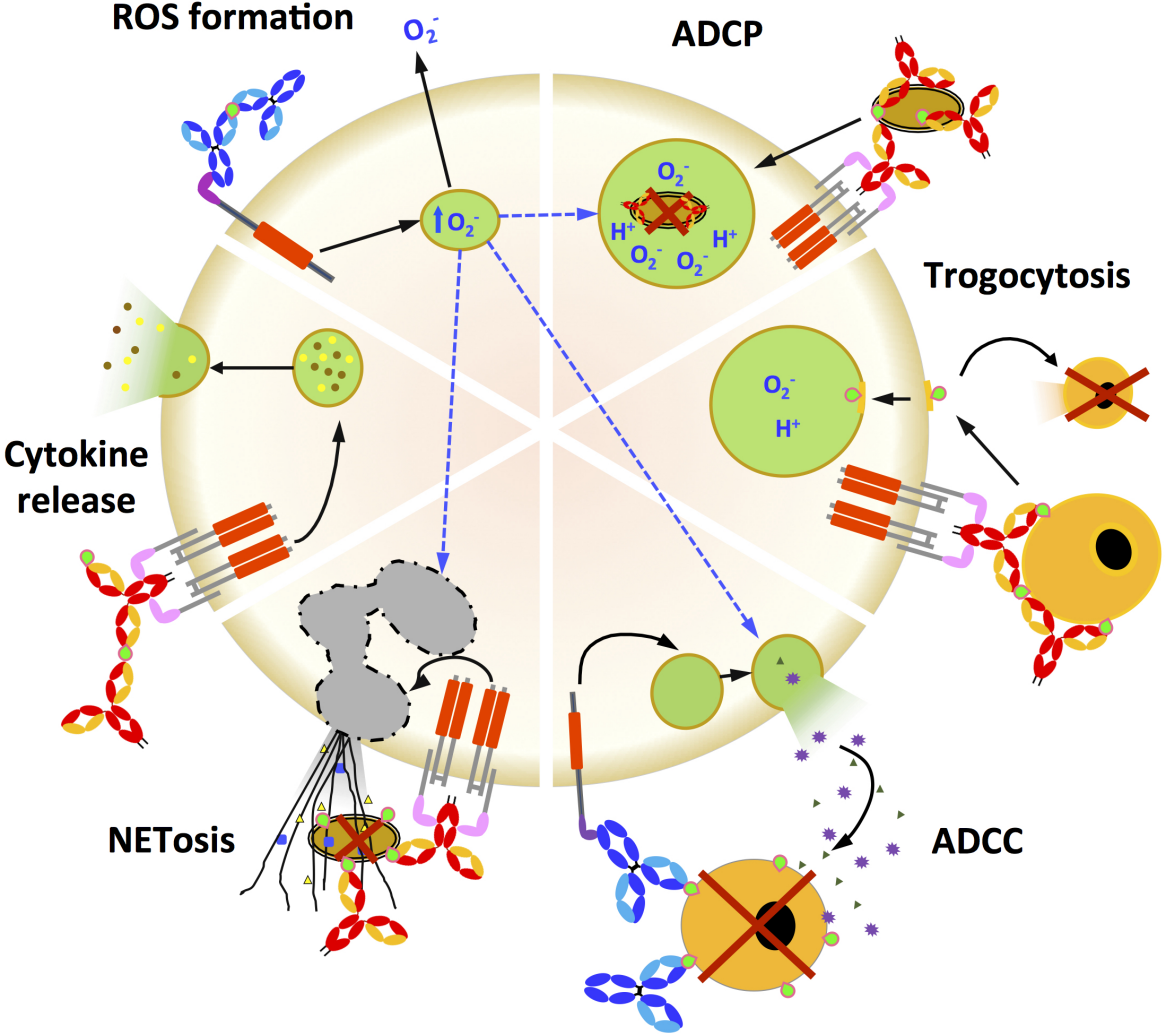
Fc-dependent effector functions triggered by FcR activation. Fc-mediated functions (ADCP, trogocytosis, ADCC, NET formation, cytokine release and ROS formation) initiated by either IgG (light and dark blue) or IgA (red and yellow) engagement with the cognate FcR that activates ITAM. IgA and IgG immune complex antigens are in green. **ADCP**: Antibody-dependent cellular phagocytosis is a potent mechanism for eliminating Ab-coated pathogens or tumour cells. Engagement of FcγR or FcαRI/CD89 expressed on innate effector cells triggers a signalling cascade leading to engulfment of the pathogen by Ab. **ADCC**: Antibody-dependent cellular cytotoxicity is a mechanism by which FcR-expressing effector cells recognise, phagocytose and kill Ab-sponsored target cells. **Trogocytosis**: It is the rapid intercellular transfer of membrane fragments and their associated molecules during intercellular contact. Trogocytosis is typically mediated by the interaction of Fc-FcRs on effector cells with opsonised target cells. Ab-dependent trogocytosis of cancer cells by neutrophils and macrophages can result in target cell death (trogocytosis-mediated cell death). **NETosis**: Neutrophil-mediated target cell death after initiation by Abs is characterised by the secretion of large neutrophil extracellular traps (NETs) that capture and destroy target cells. **Cytokine secretion**: Cross-linking of FcRs by immune complexes can induce cytokine release from innate effector cells. The type of cytokine released depends on the type of FcR stimulated. **ROS formation**: Reactive oxygen species (ROS) induced by FcR cross-linking contribute to a variety of effects such as in ADCP, ADCC and NETosis.

The integration at the effector cell level of activating and inhibitory FcR engagement after IC formation results in a wide range of functions including complement-dependent cytotoxicity (CDC), ADCC, ADCP, cytokine release, ROS production, transcytosis as well as recycling, as illustrated in **Figure 3** for FcR-mediated effector functions (8, 10). Antibody-mediated transcytosis and recycling, although crucial in Ab biology and engineering, have already been reviewed (24) and will not be discussed.

#### 2.5.1 Complement dependent cytotoxicity

The recognition by Abs of antigens, expressed on the surface of tumour or virus-infected cells, leads to their opsonisation. In turn, complement is recruited by Abs to the cell surface, forming a multi-protein complex that disrupts the lipid bilayer of the plasma membrane and kills the opsonised cell (78). Both IgG and IgA are capable of recruiting the complement system, but using different mechanisms. These differences make a direct comparison between the complement-dependent activities of the two isotypes difficult (79–81). The two pathways converge to form the terminal complement complex C5b-C9, although the underlying initiation mechanisms remain to be described.

#### 2.5.2 Antibody-dependent cellular cytotoxicity

ADCC is an effector function that relies on the interaction of Fc with an FcR on the surface of the effector cell, which triggers the destruction of the opsonised target cell (**Figure 3**). In cancer therapy, NK-dependent ADCC is one of the main mechanisms used by therapeutic Abs to eliminate cancer cells (82–84). Interestingly, monocyte-, macrophage- and neutrophil-induced ADCC is more effective when triggered by IgA- than IgG-opsonised cells. This IgA-dependent enhancement of ADCC is most likely due to the higher binding stoichiometry of Fcα to FcαRI/CD89 (13, 15, 85, 86) than that of Fcγ to FcγRI/CD64 mentioned above. In addition, IgG and IgA act synergistically to enhance lysis of HIV-1 infected cells by ADCC (85). This synergy can occur *in vivo*: indeed, the corresponding FcRs are constitutively expressed by effector cells such as primary monocytes, macrophages (85) and neutrophils (40). Of note, this is not the case in the commonly used myeloid cell line THP-1 (87).

#### 2.5.3 Antibody-dependent cell phagocytosis

Antibody-dependent cell phagocytosis can be performed by monocytes, macrophages and neutrophils stimulated by opsonised cells through activation of FcγRIIa/CD32a, FcγRI/CD64 or FcαRI/CD89, resulting in the uptake of the opsonised target cell into a phagosome (**Figure 3**). In turn, the phagosome acidifies, forming a phagolysosome in which the opsonised target cell is finally lysed (1, 41, 88). Interestingly, the presence on the effector cell surface of FcRn in addition to FcγR increases the ADCP score (59, 89), suggesting cooperation between the two classes of FcRs. Uptake of ADCP-opsonised target cells into antigen-presenting cells could result in antigen degradation, efficient cross-presentation (56, 90) and, in turn, in a specific CD8+ T-cell response (91–94). Importantly, this mechanism provides an additional direct link between humoral and cellular immunity, similar to that established by FcRn.

Only a few studies have directly compared IgG-FcγRI/CD64-mediated target cell ADCP versus IgA-FcαRI/CD89. On average, IgA induces ADCP more efficiently than IgG. HIV-1 envelope-specific IgA from a single RV144 vaccine recipient achieved robust ADCP (95), although in this study, IgG-mediated ADCP from the same individual was not studied. In addition, the HIV-1-specific broadly neutralising antibody (bNAb) 2F5 produced as an IgA isotype triggers ADCP more efficiently than when produced as an IgG isotype or compared to other anti-gp41 or -gp120 IgG bNAbs. This synergy occurs irrespective of effector cell type (46) and is consistent with other recent publications (12, 15, 16, 96–98). The few studies of IgA- versus IgG-mediated ADCP in bacterial infections have shown that monomeric IgA tends to protect humanised FcαRI/CD89 mice better against multidrug-resistant Mycobacterium tuberculosis infections than IgG, while polymeric IgA and its switched isotype IgG induce Neisseria meningitides phagocytosis in a similar manner (33, 99).

#### 2.5.4 Trogocytosis

Alternative Fc-based mechanisms for mediating Ab-dependent target cell damage have been described. Firstly, the interaction between opsonised cells and effector cells can result in the capture of a piece of the target cell membrane by the effector cell, which in most cases leads to target cell death. This mechanism is called trogocytosis (**Figure 3**). All FcγR engagements, with the exception of FcγRIIc/CD32c engagement by IgG, result in trogocytosis of opsonised cells by effector monocytes, macrophages and granulocytes (100–103). The relative efficiencies of trogocytosis mediated by different IgG subclasses have not been studied. Furthermore, the ability of mucosal IgA to induce trogocytosis is poorly described and comparative studies between isotypes are lacking (104).

#### 2.5.5 NETosis

Secondly, the formation of nuclear extracellular traps (NETs) results mainly from neutrophil-mediated cell death (**Figure 3**). NETs are formed after neutrophil activation that triggers the secretion of antimicrobial granular peptides and the release of chromatin from the cell nucleus, which together form extracellular fibres in a process called NETosis (105, 106). NETosis is emerging as an additional tool in the arsenal of antimicrobial and anticancer strategies. NETosis can be induced by both IgG and IgA, although IgA is the strongest inducer (12, 107, 108). The ability of mucosal IgA to induce NETosis remains to be tested.

#### 2.5.6 ROS formation

FcR cross-linking can stimulate the formation of intracellular ROS, which mediate a plethora of functions (**Figure 3**), including access of opsonised cells to phagosomes, NET formation followed by degranulation, and release of extracellular ROS (50, 106, 109). Thus, intracellular ROS act as second messengers to amplify ADCP and NETosis. Both IgG and IgA have been shown to stimulate ROS formation. However, IgA induces more ROS than IgG promoting NET, ADCC and ADCP (12).

#### 2.5.7 Cytokine production

Finally, cross-linking of the FcR by Abs in the IC also stimulates cytokine release from the innate effector cell (**Figure 3**). The profile of secreted cytokines depends on the type of FcR stimulated and the resulting signalling. Pro-inflammatory FcRs, namely FcγRI/CD64, FcγRIIa/CD32a, FcγRIIc/CD32c, FcγRIIIa/CD16a, FcγRIIIb/CD16b, and FcαRI/CD89, induce the release of pro-inflammatory cytokines, including IL-6, IL-2, IL-12p40, IFNγ and TNFα, while anti-inflammatory FcRs, including FcγRIIb/CD32b, will trigger the release of anti-inflammatory cytokines, such as IL-10, or block the secretion of pro-inflammatory cytokines (110–113). Once secreted, cytokines can modulate the engagement of Abs in the FcR through a mechanism called internal and external signalling (114). For example, stimulation of neutrophils by GM-CSF increases the binding of IgG and IgA to the corresponding FcR, but does not alter their expression levels. In turn, neutrophils are more likely to induce ADCC and ADCP in cancer cells (115, 116). This mechanism is particularly effective when GM-CSF synergises with IgA-mediated activation of FcαRI/CD89, triggering PI3K signalling and, downstream, ADCC and ADCP (117). The balance between pro- and anti-inflammatory cytokine secretion depends on the net ITAM and ITAMi signalling induced on FcαRI/CD89 after engagement of IgA-IC or uncomplexed IgA. For IgG, this balance depends on the type of receptor involved, either FcγRIIa/CD32a and c carrying ITAM or FcγRIIb/CD32b carrying ITIM (2, 42, 118, 119).

Overall, there is consensus on the IgA-mediated increase in effector functions. As the isotype differences probably rely on a higher binding stoichiometry to their corresponding FcR, the more FcRs engage Abs, the more efficiently downstream effector functions will be stimulated. With a greater ability to engage downstream ITAM activation than IgG (13, 18, 85, 120), IgA should be considered in the design of therapeutic Abs, alone or in combination with IgG, to enhance effector functions.

## 3 Chimeric antibodies containing Fcγ-Fcα enhance Fc-mediated functions: From the laboratory to the bedside

### 3.1 Increasing the affinity and binding avidity of Ab to FcRs

**Figure 4:**
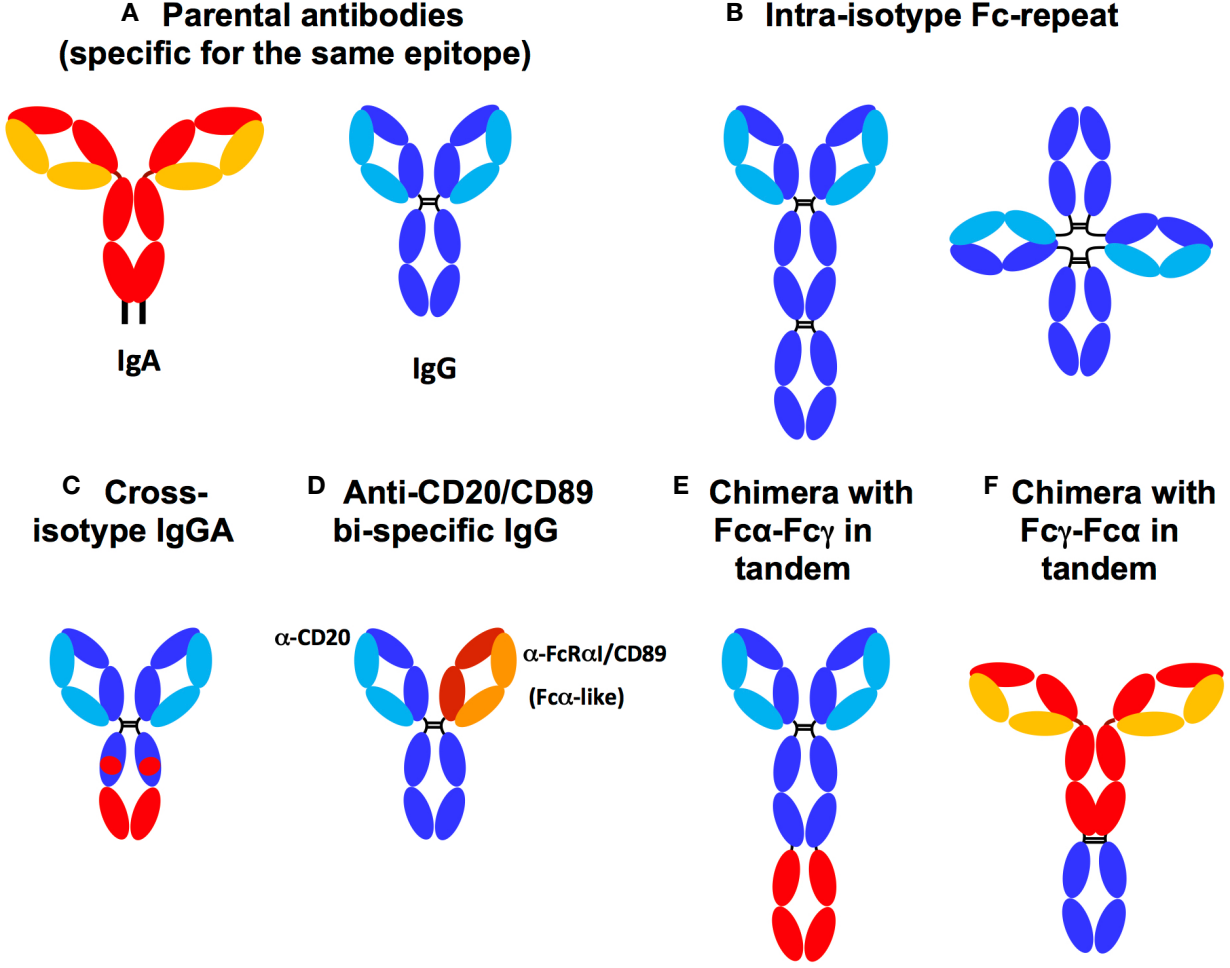
Fc chimera and parental antibodies. **(A)** Parental IgA (HC: red, LC: yellow) and IgG (HC: dark blue LC: light blue). **(B)** Fcγ duplication (dark blue) in tandem or tetrahedral form, **(C)** Cross-isotype IgGA harbouring a hybrid of Fcγ (dark blue) and Fcα (red), **(D)** Anti-CD20 and FcαRI/CD89 bi-specific IgG isotype. The chimera can bind both to FcR *via* its authentic and to FcαRI/CD89 *via* the anti-CD89 that replaces Fcα used in other constructs. **(E)** Chimera with Fcα-Fcγ expressed in tandem. **(F)** Chimera with Fcγ-Fcα expressed in tandem. Both Fcγ-Fcα and Fcα-Fcγ chimeras can harbour either an IgA2 hinge, a linker (G4S)4 or an IgG1 hinge between the two Fc chains.

Knowing that effector functions rely mainly on the Fc domains of Ab’s, the insertion of additional Fc domains into the design of Abs could result in chimeric antibodies with enhanced FcR binding and downstream Fc effector functions and therapeutic activities. To this end, from the parental Abs harboring one Fcγ (**Figure 4A**), chimeric Abs against respiratory syncytial virus (RSV) were designed with two or three Fcγ domains, resulting in a chimeric 2-Fc Ab with a tetrahedral-like geometry (121), as shown in **Figure 4B**. Surface plasmon resonance experiments showed that each 2-Fc chimera molecule binds to two FcγR molecules, thereby improving the Fc avidity of the Ab while maintaining the Fab antigen specificity of the original Ab. Each 2-Fc chimera molecule also binds to four FcRn molecules, thereby prolonging the pharmacokinetics of the chimera. However, this strategy is not optimal because significant amounts of single-chain Fc-Fab are produced along with the 2-Fc chimera. The authors did not evaluate whether increasing the avidity of the 2-Fc chimera for FcγRs improved effector functions (121).

Furthermore, Rituximab, an anti-CD20 IgG, harbouring tandemly duplicated or triplicated Fcγs broadens the spectrum of bound FcγRs from FcγRI/CD64, the only FcγR bound by the parental IgG, to FcγRIIa/CD32a (122). Simultaneous binding of FcγRI/CD64 and FcγRIIa/CD32a results in a fivefold increase in ADCC and ADCP activities against CD20+ tumour B cells in mice *in vivo* (122, 123). Such a strategy of Fcγ chimeras has not yet been developed against infectious pathogens.

### 3.2 IgG-IgA bi-isotype chimerism

IgG-IgA bi-isotype Fc chimerism was initially explored with the so-called ‘IgGA cross-isotype’ format, in which the Fc domain of the anti-HER2 IgG1 therapeutic, Trastuzumab, was genetically engineered to acquire the ability to bind to both the FcγR and FcαR (**Figure 4C**) (124). The authors elegantly determined the residues controlling the engagement of Fcα and γ with their cognate FcRs by first using alanine scanning approaches. As a result, the amino acids CHγ_1_2 and CHγ_1_3, which are not responsible for IgG engagement with the FcγR, were replaced with the corresponding amino acids of CHα_1_2 and CHα_1_3 responsible for IgA binding to the FcαRI/CD89. The resulting Trastuzumab IgGA chimera contained both Fcα and Fcγ elements. This cross-isotype IgGA chimera was well produced and had an affinity for FcγRs comparable to that of the parental IgG, although the affinity for FcαRI/CD89 decreased by two compared to the parental IgA. In addition, the affinity for C1q increased compared to parental Trastuzumab IgG and IgA. Due to its ability to bind Fcα- and FcγR, the IgGA-Trastuzumab chimera mediated higher ADCC and ADCP than the parental IgG, but did not reach the level mediated by Trastuzumab-IgA. Furthermore, the cross-isotype format showed a higher CDC compared to IgG and IgA isotypes (124). Unexpectedly, Fc engineering in the IgGA chimera abolished commitment to FcγRIIIa/CD16a and FcRn, suggesting low bioavailability *in vivo* (24). In this case, isotypic cross-transformation may therefore decrease therapeutic benefit. This study indicates that mutations focusing exclusively on amino acids directly involved in the interaction with the FcR are not functionally effective: such point mutations would probably be too targeted and unable to preserve the overall structure of each Fc domain essential for FcR engagement by Fcα and Fcγ together. Instead, it can be hypothesised that expression of the full Fcα and Fcγ is necessary to endow a chimeric Ab with a true gain of ADCC and ADCP effector functions accompanied by extended pharmacokinetics through enhanced FcRn engagement.

To enable an IgG to target FcαRI/CD89 in addition to FcγR (using its Fcγ), bi-specific IgGs with one Fab specific for FcαRI/CD89 have been designed. An example of this strategy is given by TrisoMab, a bispecific IgG (more precisely IgG1) directed against both EGFR and FcαRI/CD89 (14). This chimera offers the advantage of targeting three distinct molecules (**Figure 4D**). Indeed, TrisoMab recognises EGFR, the target of the parental IgG, *via* one Fab, as well as FcαRI/CD89 *via* the second Fab, while retaining its ability to engage FcγR *via* the Fcγ antibody (14). Consequently, TrisoMab binds to effector cells, namely neutrophils, NKs and macrophages, in turn amplifying effector functions *in vitro* and *in vivo*. As a result, neutrophils were recruited more efficiently to tumour colonies, showed improved TrisoMab-mediated ADCC and trogocytosis of cancer cells compared to the parental EGFR-specific IgG, However, the improvement over EGFR-specific IgA, whose Fab targets EGFR and own Fcα targets FcαRI/CD89, was limited. The enhancement of trogocytosis by TrisoMab may have resulted in cross-presentation of tumour antigen allowing the generation of therapeutically beneficial cytotoxic CD8+ T cell activity. In addition, macrophages showed an increase in TrisoMab-mediated tumour cell ADCP and ADCC compared to EGFR-specific IgG and IgA. Finally, in mice humanised for CD89, TrisoMab showed a prolonged half-life that was double that of EGFR-specific IgA, although similar to the IgG isoform. Improved survival and smaller tumour size in an *hCD89+* mouse tumour model (14) demonstrated a therapeutic gain of the chimera over the parental EGFR-specific IgG and IgA. These results illustrate the chief role of the IgA-FcαRI/CD89 axis in Ab-based therapy.

As this chimera lacked an Fcα domain, the true stoichiometry of an Fcα activating two FcαRI/CD89 molecules was not met and rather limited to the activation of a single FcαRI/CD89 molecule (targeted by the TrisoMab anti-CD89 Fab. This reduced engagement of FcαRI/CD89 by TrisoMab could not take advantage of the regular 1:2 IgA : FcαRI/CD89 stoichiometry. This may therefore have limited the role of FcαRI/CD89-mediated effector functions. Nevertheless, although not bearing a *bona fidae* bi-isotype Fc, a TrisoMab molecule activated both FcγRI/CD64 *via* its Fcγ and FcαRI/CD89, using an anti-CD89 Fab instead of an Fcα, with a clear and significant therapeutic advantage. The lower ratio of one chimeric TrisoMab to one FcαRI/CD89 compared to that of two Fcα, present in a single IgA molecule, to one FcαRI/CD89 may have limited the potential benefit of IgG-IgA bi-isotype chimerism. It may also explain the lack of complete remission in treated mice. It would therefore be interesting to study a tandem Fcα-Fcγ chimerism combining both Fcγ and Fcα in the same molecule. Such a chimeric Ab would increase the chimeric:FcR binding ratio to 1:3 by simultaneous binding of one FcγRI/CD64 by Fcγ and two FcαRI/CD89 by Fcα, compared to a ratio of one parental IgG to one FcγR and one parental IgA to two FcαRI/CD89. Such a chimera would be expected to increase net ITAM signalling due to the engagement of two FcαRI/CD89s in addition to the pre-existing FcγRI/CD64 (13), thereby enhancing effector functions. Again, no studies using such a bi-isotype Fc chimera enhancing FcαRI/CD89 signalling have been evaluated against infectious diseases.

To overcome these limitations, Borrok et al. designed a novel chimera, the anti-HER2 IgG1 Trastuzumab, in which the Fcα (specifically Fcα2) and its corresponding hinge were fused into the C-terminus of the Fcγ of the original IgG (17), as shown in **Figure 4E**. The chimera was produced at a level comparable to IgG and three to four times higher than Trastuzumab-IgA. The affinity of the chimera for each FcγR and FcαRI was comparable with only a slight decrease in C1q affinity compared to parental IgG1. After engagement of both FcγRI/CD64 and FcαRI/CD89 on effector cells, the chimera enhanced ADCC and ADCP of human polymorphonuclear cell-mediated tumour cells and macrophages compared to parental IgG and IgA. In addition, the chimera maintained a similar half-life to that of the parental IgG when injected intravenously into mice. In contrast, that of IgA injected into parallel animals rapidly decreased.

This important study revealed that the presence of human full-length Fcα and Fcγ in tandem in the same chimeric Ab improved Fcα- and Fcγ-mediated functions (17). The limited pharmacokinetics observed in this study could be due to the specie differences between human Ab and mouse FcR in this mouse model, probably in the glycosylation pattern of Fc. Indeed, Fc glycosylation is known to affect both effector functions and half-life of Abs (16, 125, 126). With this in mind, Li et al. developed a Rituximab chimera containing both human Fcα and Fcγ. Then, they evaluated Rituximab efficacy *in vivo* using Tg mice expressing hFcαRI/CD89 under the control of a CD14 promoter to target myeloid expression (18). In this study, the construct had the Fcγ connected to the Fcα with a polyglycine linker, with both Fc’s expressed in tandem. Functionally, in a mouse tumour model, the chimera mediated ADCC in tumour cells at levels similar to parental IgA, but greatly increased compared to parental IgG. Furthermore, when treated with the chimera, Tg *hFcαRI/CD89* mice had a smaller tumour volume compared to treatment with parental IgG or IgA. All these functional consequences were entirely dependent on hFcαRI/CD89 expression, as no effect was observed in wild-type mice (18).

Taken together, these results support the development of bi-isotypic Fc-chimeric therapeutic Abs with enhanced effector functions. Such enhancement relies on the presence of full-length Fcα and Fcγ. It should be noted that the role of the IgA hinge region may not affect functional efficacy (17, 18). However, Fc-chimeric Abs in infectious diseases have not been evaluated.

### 3.3 Fc chimeras: Remaining questions and perspectives

**Figure 5:**
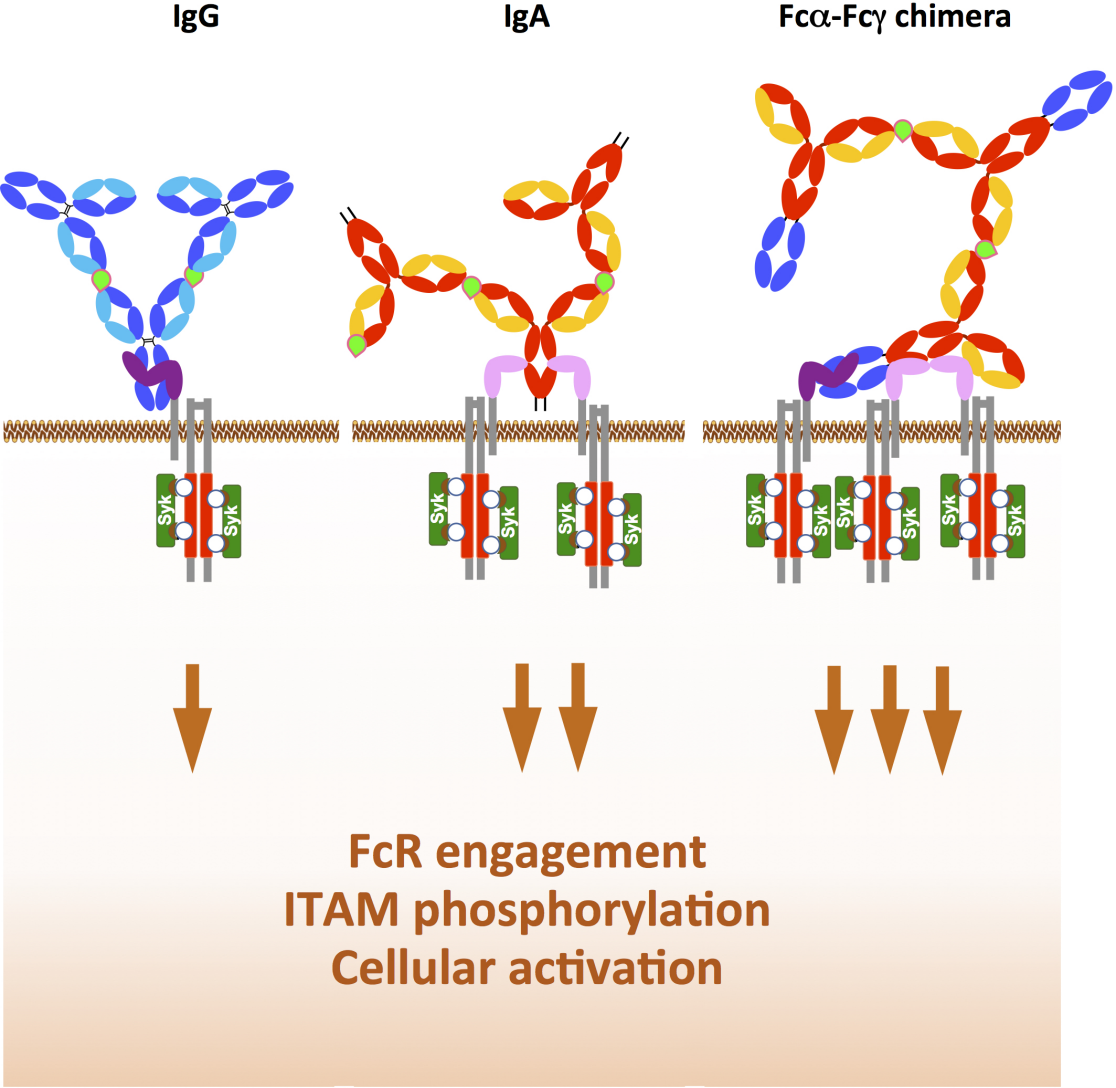
Rationale for the development of chimeras with Fcα and Fcγ expressed in tandem. While IgG in immune complex with antigen (light green) binds only to one FcγRI/CD64 (left) and the IgA immune complex binds to two FcαRI/CD89 (middle), the Fcα-Fcγ chimera immune complex binds simultaneously to one FcγRI/CD64 and two FcαRI/CD89, This increases downstream ITAM (red) phosphorylation (white circle) and subsequent Syk recruitment (dark green), thereby enhancing the functions of immune effector cells such as ADCC and ADCP.

The design of bi-isotype Fcα and Fcγ chimeras is very promising and could offer a powerful tool to target tumour cells using their enhanced Fc effector functions, especially in patients with very aggressive or resistant tumours. This anticipated enhancement would rely on increased affinity/avidity of the chimeras for their cognate FcRs and the subsequent increase in downstream signalling. This increased signalling would occur (via the ITAM domain of the FcRγ chain (paired with FcγRI/CD64 and FcαRI/CD89) or the FcR itself (for FcγRIIa/CD32a or FcγRIIc/CD32c). The total phosphorylation of ITAM induced by the chimera compared to parental IgG and IgA is expected to increase Syk recruitment to ITAM, subsequent cellular activation and effector functions, as illustrated in **Figure 5**.

Production remains an important limiting criterion in the development of such chimeric constructs. However, according to the literature, the chimeras are produced at a good level, at least comparable to that of the parental IgG. The role of the position of Fcα in the IgG/IgA chimeric Ab on effector functions remains to be explored. It could affect the respective binding of FcRs and, consequently, their ADCC and ADCP levels. Furthermore, the role of the Fab isotype, including that of the CH1 domain, also remains a parameter to be studied. In this respect, we revealed that the CH1 domain plays a crucial role in Fab antigen recognition (both in affinity and specificity) with a superiority of CH1α over CH1γ (127). Surprisingly, the higher molecular weight of Fcα-Fcγ or Fcγ-Fcγ tandem chimeras compared to parental Abs was not taken into account in these studies, probably introducing a bias. Indeed, comparing the impact of chimerism on effector functions between the chimeras and the parental IgG and IgA tested at the same molar concentration would be more rigorous than testing them at the same weight/volume concentration.

Glycoengineering is another popular method currently used to improve the functions of anti-tumour Abs, although it is mainly limited to the IgG isotype (84, 128). The impact of the glycosylation pattern of Fcα and Fcγ in chimeric Abs on therapeutic benefit remains to be investigated (86, 125) as IgA has both N- and O-glycosylations (3) and IgG has only N-glycosylations. However, the O-glycosylations of the IgA1 hinge may be too far from the CH2-CH3 domain to affect IgA interaction with FcαRI/CD89 and downstream effector functions. The impact of IgA glycosylation on Fc-mediated functions has been poorly studied. Therefore, a potential role for IgA glycosylation in the functions of Fcα-Fcγ chimeric Ab is difficult to predict. Nevertheless, desialylation of IgA1, which has been shown to enhance its pro-inflammatory properties (107), could improve the therapeutic benefits of Fcα-Fcγ chimeric Abs. However, this study did not address the impact of defucosylation on IgA-mediated effector functions. In contrast for IgG, such glycoengineering is a very promising approach (84).

Finally, the addition of an Fcα to therapeutic Abs could enhance the delivery of chimeric Ab to mucosal surfaces *via* Dectin-1, an alternative IgA receptor (129), and/or FcRn (58) capable of reversing Ab transcytosis in mucosal tissues.

### 3.4 Therapeutic Fcα-Fcγ antibodies as future anti-HIV-1 bNAbs

Given the role of the IgA isotype against viruses on the mucosal surface where FcαRI/CD89 is specifically expressed (40), we hypothesise that such tandem Fcα-Fcγ chimeric Ab (IgA with a fused Fcγ, **Figure 4F**) would be effective in the prevention of infectious pathogens such as sexually transmitted HIV-1 (130). Indeed, over the past decade, anti-HIV-1 bNAbs have been developed to either protect from infection or cure HIV-1 infected patients using passive Ab therapy (alone or in combination with antiretroviral therapy) (131). As with anti-cancer drugs, anti-HIV-1 antibodies have been engineered mainly by Fab domain engineering to improve affinity for the viral antigen and to diversify antibody specificity by using bi- or tri-specific antibodies (57, 126, 132–135). Again, Fc engineering has rarely been studied.

Despite recent advances in the beneficial role of anti-HIV-1 IgA at the mucosal level (20, 120, 129, 136–139), IgA is routinely overlooked in anti-HIV-1 immunotherapy. As an additional argument for the use of IgA, others and we have shown that IgG and IgA cooperate in effector functions *in vitro* (85, 97) and *in vivo* in the protection of vaccinated animals against repeated mucosal challenges (20, 120, 129, 136–139) as well as in the protection of vaccinated individuals as in the RV144 phase III HIV-1 clinical trial (21, 22). From these studies, it can be suggested that Fcα-Fcγ tandem chimeras engineered from bNAbs would be beneficial in protecting against mucosal acquisition of HIV-1 by triggering FcαRI/CD89 and FcγRI/CD64 signalling, thereby enhancing effector functions (**Figure 5**). This approach would complement the inhibition of mucosal entry through transcytosis and virus neutralisation. These antiviral activities are essential in the protection against sexual acquisition of HIV-1. Hence, eradication of the virus is not yet possible once infection and the resulting viral reservoir are established (20, 120, 129, 136–140). Such chimeric Abs could enhance ADCC involved in protection but also ADCP, NETosis and cytokine secretion that contribute to the establishment/maintenance of a protective immune response (20, 45, 120, 129, 136–139, 141). In addition, bi-isotype Fc chimeras could contribute, in combination with other drugs/approaches, to shock and kill strategies aimed at eliminating the HIV-1 reservoir in HIV-infected patients on suppressive antiretroviral therapy. The combination of Fab engineering and tandem Fcα-Fcγ in a single chimera could be superior to specific bi/tri constructs and tandem Fcα-Fcγ chimera alone.

Fcα-Fcγ chimeric Abs can also interact with FCRL4 and 5 on B cells to promote a memory phenotype (69, 71, 72). We have previously shown that human penile mucosa B cells express both FCRL4 and FCRL5 (142). We can suggest that during passive infusion in HIV-infected patients, Fcα-Fcγ tandem chimeras may stimulate mucosal memory B cells, and in turn plasma cells secreting protective anti-HIV-1 IgA. The latter are associated with the prevention of mucosal acquisition of HIV-1 in actively and passively vaccinated macaques and in Highly Exposed Sero-Negatif Individuals that resist to HIV infection despite unprotected sexual intercourses (19, 20, 120). Furthermore, by binding to FCRL3, Fcα-Fcγ chimeras formulated as secretory-type IgA can induce a shift from a Treg to a Th17 profile (65). The latter could contribute to the eradication of HIV reservoirs, as well as to the promotion of tumour invasion by immune effector cells, resulting in tumour clearance (65). However, sIgA cannot bind to FcαRI/CD89 and the chimerism relevant for direct effector functions could be lost in such constructs (31). Nevertheless, FCRL3-5 engagement could also lead to inhibition of B-cell functions, as described elsewhere (143–145). However, the clinical relevance of binding of therapeutic Abs to FCRL3-5 on B and T cells has not been studied. Therefore, the prediction of the biological effects of these chimeras remains uncertain. Furthermore, as FCRL6, an FcRL expressed on NK cells, cannot bind Abs, it will not interact with chimeric Abs to mediate ADCC by NKs (146).

Finally, the production of chimeric Abs could help to improve the half-life. In particular, the production of anti-HIV-1 IgG using adeno-associated vectors in mouse models prolongs its half-life compared to *in vitro* production in CHO cells (126). As the same coding sequence was used in both systems, this effect may depend on the glycosylation profile of Ab. This strategy could be beneficial for improving the half-life and effector functions of therapeutic HIV-targeted bNAbs, although the packaged coding sequence must be very compact (<4.4kb).

## 4 Conclusions and perspectives

The development of chimeric Fcα-Fcγ Abs as drugs still needs improvement, and several unexplored questions remain. The most difficult one concerns the best route of administration for the most relevant bio-distribution and stability to improve clinical efficacy. Secondly, the role of the Fcα isotype, i.e. IgA1- or IgA2-derived Fcα, in enhancing effector functions needs to be deciphered (147). Similarly, the role of IgG1- versus IgG3-derived Fcγ in these chimeras is questionable, with IgG3 being more pro-inflammatory than IgG1, although this relies on the IgG3 hinge (148). The pharmacokinetics should also be evaluated in NHPs to validate the results obtained in mouse models, as well as their half-life at different anatomical sites after intravenous or mucosal applications. Furthermore, we propose that these Fc-based chimeric Abs could offer a more effective treatment against mucosal infectious pathogens, and the generation of a higher CD8+ T cell memory response, providing vaccine-like properties to the chimera. In the case of HIV-1, these Fc-based chimeric Abs would overcome the viral resistance resulting from antiretroviral drugs and offer less toxic alternatives to HIV/AIDS patients on antiretroviral therapy. Advances in Ab engineering, probably assisted by the development of algorithms, undoubtedly rely on the constant updating of structure-function relationships of Abs. Finally, there is an increasing need to develop high-throughput screening strategies for the evaluation of Ab efficacy and the improvement of associated production pathways. All these features should encourage and stimulate the development of Ab drugs, the next challenge in immunotherapy.

## Author contributions

AC-C, DT and MB wrote and edited the manuscript. All authors contributed to the article and approved the submitted version.

## References

[1] Vidarsson G Dekkers G Rispens T. IgG subclasses and allotypes: From structure to effector functions. Front Immunol (2014) 5:520. doi: 10.3389/fimmu.2014.00520 25368619 PMC4202688

[2] Heineke MH van Egmond M. Immunoglobulin a: magic bullet or Trojan horse? Eur J Clin Invest (2017) 47:184–92. doi: 10.1111/eci.12716 28024097

[3] Mattu TS Pleass RJ Willis AC Kilian M Wormald MR Lellouch AC. The glycosylation and structure of human serum IgA1, fab, and fc regions and the role of n-glycosylation on fcα receptor interactions. J Biol Chem (1998) 273:2260–72. doi: 10.1074/jbc.273.4.2260 9442070

[4] Li F Vijayasankaran N Shen A Kiss R Amanullah A. Cell culture processes for monoclonal antibody production. MAbs (2010) 2:466–79. doi: 10.4161/mabs.2.5.12720. Yijuan. PMC2958569 20622510

[5] Wurm FM. Production of recombinant protein therapeutics in cultivated mammalian cells. Nat Biotechnol (2004) 22:1393–8. doi: 10.1038/nbt1026 15529164

[6] Chintalacharuvu KR Gurbaxani B Morrison SL. Incomplete assembly of IgA2m(2) in Chinese hamster ovary cells. Mol Immunol (2007) 44:3445–52. doi: 10.1016/j.molimm.2006.12.030 17467056

[7] Presta LG. Antibody engineering. Curr Opin Struct Biol (1992) 2:593–6. doi: 10.1016/0959-440X(92)90091-K 12948783

[8] Breedveld A van Egmond M. IgA and FcαRI: Pathological roles and therapeutic opportunities. Front Immunol (2019) 10:553. doi: 10.3389/fimmu.2019.00553 30984170 PMC6448004

[9] Ben Mkaddem S Benhamou M Monteiro RC. Understanding fc receptor involvement in inflammatory diseases: From mechanisms to new therapeutic tools. Front Immunol (2019) 10:811. doi: 10.3389/fimmu.2019.00811 31057544 PMC6481281

[10] de Taeye SW Rispens T Vidarsson G. The ligands for human IgG and their effector functions. Antibodies (2019) 8:30. doi: 10.3390/antib8020030 31544836 PMC6640714

[11] Carter PJ Lazar GA. Next generation antibody drugs: Pursuit of the “high-hanging fruit”. Nat Rev Drug Discovery (2018) 17:197–223. doi: 10.1038/nrd.2017.227 29192287

[12] Aleyd E van Hout MWM Ganzevles SH Hoeben KA Everts V Bakema JE. IgA enhances NETosis and release of neutrophil extracellular traps by polymorphonuclear cells *via* fcα receptor I. J Immunol Baltim Md (2014) 1950:192. doi: 10.4049/jimmunol.1300261 24493821

[13] Brandsma AM Bondza S Evers M Koutstaal R Nederend M Jansen JHM. Potent fc receptor signaling by IgA leads to superior killing of cancer cells by neutrophils compared to IgG. Front Immunol (2019) 10:704. doi: 10.3389/fimmu.2019.00704 31031746 PMC6470253

[14] Heemskerk N Gruijs M Temming AR Heineke MH Gout DY Hellingman T. Augmented antibody-based anticancer therapeutics boost neutrophil cytotoxicity. J Clin Invest (2021) 131:134680. doi: 10.1172/JCI134680 33561014 PMC7954609

[15] Lohse S Loew S Kretschmer A Jansen JHM Meyer S Broeke T t. Effector mechanisms of IgA antibodies against CD20 include recruitment of myeloid cells for antibody-dependent cell-mediated cytotoxicity and complement-dependent cytotoxicity. Br J Haematol (2018) 181:413–7. doi: 10.1111/bjh.14624 28449349

[16] Lohse S Meyer S Meulenbroek LAPM Jansen JHM Nederend M Kretschmer A. An anti-EGFR IgA that displays improved pharmacokinetics and myeloid effector cell engagement in vivo. Cancer Res (2016) 76:403–17. doi: 10.1158/0008-5472.CAN-15-1232 26634925

[17] Borrok MJ Luheshi NM Beyaz N Davies GC Legg JW Wu H. Enhancement of antibody-dependent cell-mediated cytotoxicity by endowing IgG with FcαRI (CD89) binding. mAbs (2015) 7:743–51. doi: 10.1080/19420862.2015.1047570 PMC4623516 25970007

[18] Li B Xu L Tao F Xie K Wu Z Li Y. Simultaneous exposure to FcγR and FcαR on monocytes and macrophages enhances antitumor activity in vivo. Oncotarget (2017) 8:39356. doi: 10.18632/oncotarget.17000 28454118 PMC5503618

[19] Tudor D Derrien M Diomede L Drillet A-S Houimel M Moog C. HIV-1 gp41-specific monoclonal mucosal IgAs derived from highly exposed but IgG-seronegative individuals block HIV-1 epithelial transcytosis and neutralize CD4+ cell infection: an IgA gene and functional analysis. Mucosal Immunol (2009) 2:412–26. doi: 10.1038/mi.2009.89 19587640

[20] Bomsel M Tudor D Drillet A-S Alfsen A Ganor Y Roger M-G. Immunization with HIV-1 gp41 subunit virosomes induces mucosal antibodies protecting nonhuman primates against vaginal SHIV challenges. Immunity (2011) 34:269–80. doi: 10.1016/j.immuni.2011.01.015 21315623

[21] Gong S Lakhashe SK Hariraju D Scinto H Lanzavecchia A Cameroni E. Cooperation between systemic IgG1 and mucosal dimeric IgA2 monoclonal anti-HIV env antibodies: Passive immunization protects Indian rhesus macaques against mucosal SHIV challenges. Front Immunol (2021) 12:705592. doi: 10.3389/fimmu.2021.705592 34413855 PMC8370093

[22] Fischinger S Dolatshahi S Jennewein MF Rerks-Ngarm S Pitisuttithum P Nitayaphan S. IgG3 collaborates with IgG1 and IgA to recruit effector function in RV144 vaccinees. JCI Insight (2020) 5:e140925. doi: 10.1172/jci.insight.140925 33031099 PMC7710302

[23] Pincetic A Bournazos S DiLillo DJ Maamary J Wang TT Dahan R. Type I and type II fc receptors regulate innate and adaptive immunity. Nat Immunol (2014) 15:707–16. doi: 10.1038/ni.2939 PMC7430760 25045879

[24] Pyzik M Sand KMK Hubbard JJ Andersen JT Sandlie I Blumberg RS. The neonatal fc receptor (FcRn): A misnomer? Front Immunol (2019) 10:1540. doi: 10.3389/fimmu.2019.01540 31354709 PMC6636548

[25] Foss S Bottermann M Jonsson A Sandlie I James LC Andersen JT. TRIM21-from intracellular immunity to therapy. Front Immunol (2019) 10:2049. doi: 10.3389/fimmu.2019.02049 31555278 PMC6722209

[26] Herr AB Ballister ER Bjorkman PJ. Insights into IgA-mediated immune responses from the crystal structures of human FcαRI and its complex with IgA1-fc. Nature (2003) 423:614–20. doi: 10.1038/nature01685 12768205

[27] Bonner A Furtado PB Almogren A Kerr MA Perkins SJ. Implications of the near-planar solution structure of human myeloma dimeric IgA1 for mucosal immunity and IgA nephropathy. J Immunol (2008) 180:1008–18. doi: 10.4049/jimmunol.180.2.1008 18178841

[28] Correa A Trajtenberg F Obal G Pritsch O Dighiero G Oppezzo P. Structure of a human IgA1 fab fragment at 1.55 Å resolution: Potential effect of the constant domains on antigen-affinity modulation. Acta Crystallogr D Biol Crystallogr (2013) 69:388–97. doi: 10.1107/S0907444912048664 23519414

[29] Kerr MA. The structure and function of human IgA. Biochem J (1990) 271:285–96. doi: 10.1042/bj2710285 PMC1149552 2241915

[30] Getahun A Cambier JC. Of ITIMs, ITAMs, and ITAMis: Revisiting immunoglobulin fc receptor signaling. Immunol Rev (2015) 268:66–73. doi: 10.1111/imr.12336 26497513 PMC4621791

[31] Carayannopoulos L Hexham JM Capra JD. Localization of the binding site for the monocyte immunoglobulin (Ig) a-fc receptor (CD89) to the domain boundary between Calpha2 and Calpha3 in human IgA1. J Exp Med (1996) 183:1579–86. doi: 10.1084/jem.183.4.1579 PMC2192530 8666916

[32] van der Steen L Tuk CW Bakema JE Kooij G Reijerkerk A Vidarsson G. Immunoglobulin a: Fc(alpha)RI interactions induce neutrophil migration through release of leukotriene B4. Gastroenterology (2009) 137:2018–2029.e1–3. doi: 10.1053/j.gastro.2009.06.047 19555692

[33] Vidarsson G van der Pol W-L van den Elsen JMH Vilé H Jansen M Duijs J. Activity of human IgG and IgA subclasses in immune defense against neisseria meningitidis serogroup b. J Immunol (2001) 166:6250–6. doi: 10.4049/jimmunol.166.10.6250 11342648

[34] Kikuno K Kang D-W Tahara K Torii I Kubagawa HM Ho KJ. Unusual biochemical features and follicular dendritic cell expression of human fcα/μ receptor. Eur J Immunol (2007) 37:3540–50. doi: 10.1002/eji.200737655 PMC4160165 18000956

[35] Baumann J Park CG Mantis NJ. Recognition of secretory IgA by DC-SIGN: implications for immune surveillance in the intestine. Immunol Lett (2010) 131:59–66. doi: 10.1016/j.imlet.2010.03.005 20362001 PMC2954462

[36] Yu X Vasiljevic S Mitchell DA Crispin M Scanlan CN. Dissecting the molecular mechanism of IVIg therapy: The interaction between serum IgG and DC-SIGN is independent of antibody glycoform or fc domain. J Mol Biol (2013) 425:1253–8. doi: 10.1016/j.jmb.2013.02.006 23416198

[37] Figdor CG van Kooyk Y Adema GJ. C-type lectin receptors on dendritic cells and langerhans cells. Nat Rev Immunol (2002) 2:77–84. doi: 10.1038/nri723 11910898

[38] Kara S Amon L Lühr JJ Nimmerjahn F Dudziak D Lux A. Impact of plasma membrane domains on IgG fc receptor function. Front Immunol (2020) 11:1320. doi: 10.3389/fimmu.2020.01320 32714325 PMC7344230

[39] Temming AR Dekkers G van de Bovenkamp FS Plomp HR Bentlage AEH Szittner Z. Human DC-SIGN and CD23 do not interact with human IgG. Sci Rep (2019) 9:9995. doi: 10.1038/s41598-019-46484-2 31292524 PMC6620288

[40] Cheeseman HM Carias AM Evans AB Olejniczak NJ Ziprin P King DF. Expression profile of human fc receptors in mucosal tissue: Implications for antibody-dependent cellular effector functions targeting HIV-1 transmission. PLoS One (2016) 11:e0154656. doi: 10.1371/journal.pone.0154656 27164006 PMC4862624

[41] Tay MZ Wiehe K Pollara J. Antibody-dependent cellular phagocytosis in antiviral immune responses. Front Immunol (2019) 10:332. doi: 10.3389/fimmu.2019.00332 30873178 PMC6404786

[42] Bournazos S Wang TT Dahan R Maamary J Ravetch JV. Signaling by antibodies: Recent progress. Annu Rev Immunol (2017) 35:285–311. doi: 10.1146/annurev-immunol-051116-052433 28446061 PMC5613280

[43] Lundberg K Rydnert F Broos S Andersson M Greiff L Lindstedt M. Allergen-specific immunotherapy alters the frequency, as well as the FcR and CLR expression profiles of human dendritic cell subsets. PLoS One (2016) 11:e0148838. doi: 10.1371/journal.pone.0148838 26863539 PMC4749279

[44] Shen R Richter HE Clements RH Novak L Huff K Bimczok D. Macrophages in vaginal but not intestinal mucosa are monocyte-like and permissive to human immunodeficiency virus type 1 infection. J Virol (2009) 83:3258–67. doi: 10.1128/JVI.01796-08 PMC2655566 19153236

[45] Sips M Krykbaeva M Diefenbach TJ Ghebremichael M Bowman BA Dugast A-S. Fc receptor-mediated phagocytosis in tissues as a potent mechanism for preventive and therapeutic HIV vaccine strategies. Mucosal Immunol (2016) 9:1584–95. doi: 10.1038/mi.2016.12 PMC4988947 26883728

[46] Duchemin M Tudor D Cottignies-Calamarte A Bomsel M. Antibody-dependent cellular phagocytosis of HIV-1-Infected cells is efficiently triggered by IgA targeting HIV-1 envelope subunit gp41. Front Immunol (2020) 11:1141. doi: 10.3389/fimmu.2020.01141 32582208 PMC7296124

[47] Oortwijn BD Roos A Royle L van Gijlswijk-Janssen DJ Faber-Krol MC Eijgenraam JW. Differential glycosylation of polymeric and monomeric IgA: A possible role in glomerular inflammation in IgA nephropathy. J Am Soc Nephrol (2006) 17:3529–39. doi: 10.1681/ASN.2006040388 17050773

[48] Roghanian A Stopforth RJ Dahal LN Cragg MS. New revelations from an old receptor: Immunoregulatory functions of the inhibitory fc gamma receptor, FcγRIIB (CD32B). J Leukoc Biol (2018) 103:1077–88. doi: 10.1002/JLB.2MIR0917-354R 29406570

[49] Davis SK Selva KJ Kent SJ Chung AW. Serum IgA fc effector functions in infectious disease and cancer. Immunol Cell Biol (2019). doi: 10.1111/imcb.12306 PMC7217208 31785006

[50] Ben Mkaddem S Hayem G Jönsson F Rossato E Boedec E Boussetta T. Shifting FcγRIIA-ITAM from activation to inhibitory configuration ameliorates arthritis. J Clin Invest (2014) 124:3945–59. doi: 10.1172/JCI74572 PMC4151227 25061875

[51] Boesch AW Brown EP Cheng HD Ofori MO Normandin E Nigrovic PA. Highly parallel characterization of IgG fc binding interactions. MAbs (2014) 6:915–27. doi: 10.4161/mabs.28808 PMC4171026 24927273

[52] Bidgood SR Tam JCH McEwan WA Mallery DL James LC. Translocalized IgA mediates neutralization and stimulates innate immunity inside infected cells. Proc Natl Acad Sci USA (2014) 111:13463–8. doi: 10.1073/pnas.1410980111 PMC4169910 25169018

[53] James LC Keeble AH Khan Z Rhodes DA Trowsdale J. Structural basis for PRYSPRY-mediated tripartite motif (TRIM) protein function. Proc Natl Acad Sci USA (2007) 104:6200–5. doi: 10.1073/pnas.0609174104 PMC1851072 17400754

[54] Caddy SL Vaysburd M Wing M Foss S Andersen JT O’Connell K. Intracellular neutralisation of rotavirus by VP6-specific IgG. PLoS Pathog (2020) 16:e1008732. doi: 10.1371/journal.ppat.1008732 32750093 PMC7428215

[55] Ng PML Kaliaperumal N Lee CY Chin WJ Tan HC Au VB. Enhancing antigen cross-presentation in human monocyte-derived dendritic cells by recruiting the intracellular fc receptor TRIM21. J Immunol (2019) 276–286. doi: 10.4049/jimmunol.1800462 30796180

[56] Liu X Lu L Yang Z Palaniyandi S Zeng R Gao L-Y. The neonatal FcR-mediated presentation of immune-complexed antigen is associated with endosomal and phagosomal pH and antigen stability in macrophages and dendritic cells. J Immunol Baltim Md 1950 (2011) 186:4674–86. doi: 10.4049/jimmunol.1003584 21402891

[57] Ko S-Y Pegu A Rudicell RS Yang Z Joyce MG Chen X. Enhanced neonatal fc receptor function improves protection against primate SHIV infection. Nature (2014) 514:642–5. doi: 10.1038/nature13612 PMC4433741 25119033

[58] Baker K Rath T Pyzik M Blumberg RS. The role of FcRn in antigen presentation. Front Immunol (2014) 5:408. doi: 10.3389/fimmu.2014.00408 25221553 PMC4145246

[59] Qiao S-W Kobayashi K Johansen F-E Sollid LM Andersen JT Milford E. Dependence of antibody-mediated presentation of antigen on FcRn. Proc Natl Acad Sci (2008) 105:9337–42. doi: 10.1073/pnas.0801717105 PMC2453734 18599440

[60] Zalevsky J Chamberlain AK Horton HM Karki S Leung IWL Sproule TJ. Enhanced antibody half-life improves *in vivo* activity. Nat Biotechnol (2010) 28:157–9. doi: 10.1038/nbt.1601 PMC2855492 20081867

[61] Mechetina LV Najakshin AM Volkova OY Guselnikov SV Faizulin RZ Alabyev BY. FCRL, a novel member of the leukocyte fc receptor family possesses unique structural features. Eur J Immunol (2002) 32:87–96. doi: 10.1002/1521-4141(200201)32:1<87 11754007

[62] Davis RS. Fc receptor-like molecules. Annu Rev Immunol (2007) 25:525–60. doi: 10.1146/annurev.immunol.25.022106.141541 17201682

[63] Davis RS Ehrhardt GRA Leu C-M Hirano M Cooper MD. An extended family of fc receptor relatives. Eur J Immunol (2005) 35:674–80. doi: 10.1002/eji.200425886 15688344

[64] Rostamzadeh D Kazemi T Amirghofran Z Shabani M. Update on fc receptor-like (FCRL) family: New immunoregulatory players in health and diseases. Expert Opin Ther Targets (2018) 22:487–502. doi: 10.1080/14728222.2018.1472768 29737217

[65] Agarwal S Kraus Z Dement-Brown J Alabi O Starost K Tolnay M. Human fc receptor-like 3 inhibits regulatory T cell function and binds secretory IgA. Cell Rep (2020) 30:1292–1299.e3. doi: 10.1016/j.celrep.2019.12.099 32023449

[66] Liu Y Goroshko S Leung LYT Dong S Khan S Campisi P. FCRL4 is an fc receptor for systemic IgA, but not mucosal secretory IgA. J Immunol Baltim Md 1950 (2020) 205:533–8. doi: 10.4049/jimmunol.2000293 32513851

[67] Wilson TJ Fuchs A Colonna M. Human FcRL4 and FcRL5 are receptors for IgA and IgG. J Immunol Baltim Md 1950 (2012) 188:4741–5. doi: 10.4049/jimmunol.1102651 PMC3634363 22491254

[68] Franco A Damdinsuren B Ise T Dement-Brown J Li H Nagata S. Human fc receptor-like 5 binds intact IgG *via* mechanisms distinct from those of fc receptors. J Immunol Baltim Md 1950 (2013) 190:5739–46. doi: 10.4049/jimmunol.1202860 PMC3660407 23616577

[69] Dement-Brown J Newton CS Ise T Damdinsuren B Nagata S Tolnay M. Fc receptor-like 5 promotes b cell proliferation and drives the development of cells displaying switched isotypes. J Leukoc Biol (2012) 91:59–67. doi: 10.1189/jlb.0211096 22028333

[70] King HW Orban N Riches JC Clear AJ Warnes G Teichmann SA. Single-cell analysis of human b cell maturation predicts how antibody class switching shapes selection dynamics. Sci Immunol (2021) 6:eabe6291. doi: 10.1126/sciimmunol.abe6291 33579751

[71] Li H Dement-Brown J Liao P-J Mazo I Mills F Kraus Z. Fc receptor-like 4 and 5 define human atypical memory b cells. Int Immunol (2020) 32:755–70. doi: 10.1093/intimm/dxaa053 32805738

[72] Tolnay M. Lymphocytes sense antibodies through human FCRL proteins: Emerging roles in mucosal immunity. J Leukoc Biol (2022) 111:477–87. doi: 10.1002/JLB.4RU0221-102RR 33884658

[73] Akula S Mohammadamin S Hellman L. Fc receptors for immunoglobulins and their appearance during vertebrate evolution. PLoS One (2014) 9:e96903. doi: 10.1371/journal.pone.0096903 24816777 PMC4016189

[74] Crowley AR Ackerman ME. Mind the gap: How interspecies variability in IgG and its receptors may complicate comparisons of human and non-human primate effector function. Front Immunol (2019) 10:697. doi: 10.3389/fimmu.2019.00697 31024542 PMC6463756

[75] Dekkers G Bentlage AEH Stegmann TC Howie HL Lissenberg-Thunnissen S Zimring J. Affinity of human IgG subclasses to mouse fc gamma receptors. MAbs (2017) 9:767–73. doi: 10.1080/19420862.2017.1323159 PMC5524164 28463043

[76] Mancardi D Daëron M. Fc receptors in immune responses. Ref Module BioMed Sci (2014). doi: 10.1016/B978-0-12-801238-3.00119-7

[77] Crowley AR Osei-Owusu NY Dekkers G Gao W Wuhrer M Magnani DM. Biophysical evaluation of rhesus macaque fc gamma receptors reveals similar IgG fc glycoform preferences to human receptors. Front Immunol (2021) 12:754710. doi: 10.3389/fimmu.2021.754710 34712242 PMC8546228

[78] Sarma JV Ward PA. The complement system. Cell Tissue Res (2011) 343:227–35. doi: 10.1007/s00441-010-1034-0 PMC3097465 20838815

[79] Itami H Hara S Samejima K Tsushima H Morimoto K Okamoto K. Complement activation is associated with crescent formation in IgA nephropathy. Virchows Arch (2020) 565–572. doi: 10.1007/s00428-020-02800-0 32300880

[80] Roos A Bouwman LH van Gijlswijk-Janssen DJ Faber-Krol MC Stahl GL Daha MR. Human IgA activates the complement system *via* the mannan-binding lectin pathway. J Immunol Baltim Md 1950 (2001) 167:2861–8. doi: 10.4049/jimmunol.167.5.2861 11509633

[81] Bindon CI Hale G Brüggemann M Waldmann H. Human monoclonal IgG isotypes differ in complement activating function at the level of C4 as well as C1q. J Exp Med (1988) 168:127–42. doi: 10.1084/jem.168.1.127 PMC2188986 3260935

[82] Miller ML Finn OJ. Chapter twenty-two - flow cytometry-based assessment of direct-targeting anti-cancer antibody immune effector functions. In: Galluzzi L Rudqvist N-P, editors. Methods in enzymology. tumor immunology and immunotherapy – cellular methods part b. Academic Press (2020). 632:431–56. doi: 10.1016/bs.mie.2019.07.026 PMC7000137 32000909

[83] Ochoa MC Minute L Rodriguez I Garasa S Perez-Ruiz E Inogés S. Antibody-dependent cell cytotoxicity: Immunotherapy strategies enhancing effector NK cells. Immunol Cell Biol (2017) 95:347–55. doi: 10.1038/icb.2017.6 28138156

[84] Pereira NA Chan KF Lin PC Song Z. The “less-is-more” in therapeutic antibodies: Afucosylated anti-cancer antibodies with enhanced antibody-dependent cellular cytotoxicity. MAbs (2018) 10:693–711. doi: 10.1080/19420862.2018.1466767 29733746 PMC6150623

[85] Duchemin M Khamassi M Xu L Tudor D Bomsel M. IgA targeting human immunodeficiency virus-1 envelope gp41 triggers antibody-dependent cellular cytotoxicity cross-clade and cooperates with gp41-specific IgG to increase cell lysis. Front Immunol (2018) 9:244. doi: 10.3389/fimmu.2018.00244 29651286 PMC5884934

[86] Treffers LW ten Broeke T Rösner T Jansen JHM van Houdt M Kahle S. IgA-mediated killing of tumor cells by neutrophils is enhanced by CD47–SIRPα checkpoint inhibition. Cancer Immunol Res (2020) 8:120–30. doi: 10.1158/2326-6066.CIR-19-0144 31690649

[87] Tay MZ Liu P Williams LD McRaven MD Sawant S Gurley TC. Antibody-mediated internalization of infectious HIV-1 virions differs among antibody isotypes and subclasses. PLoS Pathog (2016) 12:e1005817. doi: 10.1371/journal.ppat.1005817 27579713 PMC5007037

[88] Plotkin SA. Correlates of protection induced by vaccination. Clin Vaccine Immunol (2010) 17:1055–65. doi: 10.1128/CVI.00131-10 PMC2897268 20463105

[89] Vidarsson G Stemerding AM Stapleton NM Spliethoff SE Janssen H Rebers FE. FcRn: An IgG receptor on phagocytes with a novel role in phagocytosis. Blood (2006) 108:3573–9. doi: 10.1182/blood-2006-05-024539 16849638

[90] León B Ballesteros-Tato A Randall TD Lund FE. Prolonged antigen presentation by immune complex–binding dendritic cells programs the proliferative capacity of memory CD8 T cells. J Exp Med (2014) 211:1637–55. doi: 10.1084/jem.20131692 PMC4113940 25002751

[91] Villinger F Mayne AE Bostik P Mori K Jensen PE Ahmed R. Evidence for antibody-mediated enhancement of simian immunodeficiency virus (SIV) gag antigen processing and cross presentation in SIV-infected rhesus macaques. J Virol (2003) 77:10–24. doi: 10.1128/jvi.77.1.10-24.2003 12477806 PMC140624

[92] Niu M Wong YC Wang H Li X Chan CY Zhang Q. Tandem bispecific antibody prevents pathogenic SHIVSF162P3CN infection and disease progression. Cell Rep (2021) 36:109611. doi: 10.1016/j.celrep.2021.109611 34433029

[93] Gall VA Philips AV Qiao N Clise-Dwyer K Perakis AA Zhang M. Trastuzumab increases HER2 uptake and cross-presentation by dendritic cells. Cancer Res (2017) 77:5374–83. doi: 10.1158/0008-5472.CAN-16-2774 PMC5626640 28819024

[94] Tseng D Volkmer J-P Willingham SB Contreras-Trujillo H Fathman JW Fernhoff NB. Anti-CD47 antibody-mediated phagocytosis of cancer by macrophages primes an effective antitumor T-cell response. Proc Natl Acad Sci USA (2013) 110:11103–8. doi: 10.1073/pnas.1305569110 PMC3703977 23690610

[95] Wills S Hwang K-K Liu P Dennison SM Tay MZ Shen X. HIV-1-Specific IgA monoclonal antibodies from an HIV-1 vaccinee mediate galactosylceramide blocking and phagocytosis. J Virol (2018) 92:e01552–17. doi: 10.1128/JVI.01552-17 PMC5972890 29321320

[96] Evers M Rösner T Dünkel A Jansen JHM Baumann N ten Broeke T. The selection of variable regions affects effector mechanisms of IgA antibodies against CD20. Blood Adv (2021) 5:3807–20. doi: 10.1182/bloodadvances.2021004598 PMC8679664 34525171

[97] Brandsma AM ten Broeke T Nederend M Meulenbroek LAPM van Tetering G Meyer S. Simultaneous targeting of fc rs and fc RI enhances tumor cell killing. Cancer Immunol Res (2015) 3:1316–24. doi: 10.1158/2326-6066.CIR-15-0099-T 26407589

[98] Evers M Ten Broeke T Jansen JHM Nederend M Hamdan F Reiding KR. Novel chimerized IgA CD20 antibodies: Improving neutrophil activation against CD20-positive malignancies. mAbs (2020) 12:1795505. doi: 10.1080/19420862.2020.1795505 32744145 PMC7531568

[99] Tran AC Diogo GR Paul MJ Copland A Hart P Mehta N. Mucosal therapy of multi-drug resistant tuberculosis with IgA and interferon-γ. Front Immunol (2020) 11:582833. doi: 10.3389/fimmu.2020.582833 33193394 PMC7606302

[100] Iwasaki S Masuda S Baba T Tomaru U Katsumata K Kasahara M. Plasma-dependent, antibody- and fcγ receptor-mediated translocation of CD8 molecules from T cells to monocytes. Cytometry A (2011) 79A:46–56. doi: 10.1002/cyto.a.20984 21182182

[101] Daubeuf S Lindorfer MA Taylor RP Joly E Hudrisier D. The direction of plasma membrane exchange between lymphocytes and accessory cells by trogocytosis is influenced by the nature of the accessory cell. J Immunol (2010) 184:1897–908. doi: 10.4049/jimmunol.0901570 20089699

[102] Masuda S Iwasaki S Tomaru U Sato J Kawakami A Ichijo K. Mechanism of fcγ receptor-mediated trogocytosis-based false-positive results in flow cytometry. PLoS One (2012) 7:e52918. doi: 10.1371/journal.pone.0052918 23300821 PMC3531343

[103] Beum PV Mack DA Pawluczkowycz AW Lindorfer MA Taylor RP. Binding of rituximab, trastuzumab, cetuximab, or mAb T101 to cancer cells promotes trogocytosis mediated by THP-1 cells and monocytes. J Immunol (2008) 181:8120–32. doi: 10.4049/jimmunol.181.11.8120 19018005

[104] Chadebech P Michel M Janvier D Yamada K Copie-Bergman C Bodivit G. IgA-mediated human autoimmune hemolytic anemia as a result of hemagglutination in the spleen, but independent of complement activation and FcαRI. Blood (2010) 116:4141–7. doi: 10.1182/blood-2010-03-276162 20644119

[105] Estúa-Acosta GA Zamora-Ortiz R Buentello-Volante B García-Mejía M Garfias Y. Neutrophil extracellular traps: Current perspectives in the eye. Cells (2019) 8:979. doi: 10.3390/cells8090979 31461831 PMC6769795

[106] Fuchs TA Abed U Goosmann C Hurwitz R Schulze I Wahn V. Novel cell death program leads to neutrophil extracellular traps. J Cell Biol (2007) 176:231–41. doi: 10.1083/jcb.200606027 PMC2063942 17210947

[107] Steffen U Koeleman CA Sokolova MV Bang H Kleyer A Rech J. IgA subclasses have different effector functions associated with distinct glycosylation profiles. Nat Commun (2020) 11:120. doi: 10.1038/s41467-019-13992-8 31913287 PMC6949214

[108] Chen K Nishi H Travers R Tsuboi N Martinod K Wagner DD. Endocytosis of soluble immune complexes leads to their clearance by FcγRIIIB but induces neutrophil extracellular traps *via* FcγRIIA in vivo. Blood (2012) 120:4421–31. doi: 10.1182/blood-2011-12-401133 PMC3507149 22955924

[109] Babior BM. Phagocytes and oxidative stress. Am J Med (2000) 109:33–44. doi: 10.1016/s0002-9343(00)00481-2 10936476

[110] Mishra HK Pore N Michelotti EF Walcheck B. Anti-ADAM17 monoclonal antibody MEDI3622 increases IFNγ production by human NK cells in the presence of antibody-bound tumor cells. Cancer Immunol Immunother CII (2018) 67:1407–16. doi: 10.1007/s00262-018-2193-1 PMC6126979 29978334

[111] Gayet R Michaud E Nicoli F Chanut B Paul M Rochereau N. Impact of IgA isoforms on their ability to activate dendritic cells and to prime T cells. Eur J Immunol (2020) 50:1295–306. doi: 10.1002/eji.201948177 32277709

[112] Boross P van Montfoort N Stapels DAC van der Poel CE Bertens C Meeldijk J. FcRγ-chain ITAM signaling is critically required for cross-presentation of soluble antibody–antigen complexes by dendritic cells. J Immunol (2014) 193:5506–14. doi: 10.4049/jimmunol.1302012 25355925

[113] Siedlar M Strach M Bukowska-Strakova K Lenart M Szaflarska A Węglarczyk K. Preparations of intravenous immunoglobulins diminish the number and proinflammatory response of CD14+CD16++ monocytes in common variable immunodeficiency (CVID) patients. Clin Immunol Orlando Fla (2011) 139:122–32. doi: 10.1016/j.clim.2011.01.002 21300572

[114] Koenderman L. Inside-out control of fc-receptors. Front Immunol (2019) 10:544. doi: 10.3389/fimmu.2019.00544 30949181 PMC6437074

[115] Repp R Valerius T Sendler A Gramatzki M Iro H Kalden JR. Neutrophils express the high affinity receptor for IgG (Fc gamma RI, CD64) after *in vivo* application of recombinant human granulocyte colony-stimulating factor. Blood (1991) 78:885–9. doi: 10.1182/blood.V78.4.885.885 1714327

[116] Weisbart RH Kacena A Schuh A Golde DW. GM-CSF induces human neutrophil IgA-mediated phagocytosis by an IgA fc receptor activation mechanism. Nature (1988) 332:647–8. doi: 10.1038/332647a0 2451784

[117] Bracke M Nijhuis E Lammers JW Coffer PJ Koenderman L. A critical role for PI 3-kinase in cytokine-induced fcalpha-receptor activation. Blood (2000) 95:2037–43. doi: 10.1182/blood.V95.6.2037 10706872

[118] Hansen IS Hoepel W Zaat SAJ Baeten DLP den Dunnen J. Serum IgA immune complexes promote proinflammatory cytokine production by human macrophages, monocytes, and kupffer cells through FcαRI-TLR cross-talk. J Immunol Baltim Md 1950 (2017) 199:4124–31. doi: 10.4049/jimmunol.1700883 29118246

[119] Anthony RM Kobayashi T Wermeling F Ravetch JV. Intravenous gammaglobulin suppresses inflammation through a novel T(H)2 pathway. Nature (2011) 475:110–3. doi: 10.1038/nature10134 PMC3694429 21685887

[120] Sholukh AM Watkins JD Vyas HK Gupta S Lakhashe SK Thorat S. Defense-in-depth by mucosally administered anti-HIV dimeric IgA2 and systemic IgG1 mAbs: Complete protection of rhesus monkeys from mucosal SHIV challenge. Vaccine (2015) 33:2086–95. doi: 10.1016/j.vaccine.2015.02.020 PMC4411954 25769884

[121] Goulet DR Zwolak A Williams JA Chiu ML Atkins WM. Design and characterization of novel dual fc antibody with enhanced avidity for fc receptors. Proteins (2020) 88:689–97. doi: 10.1002/prot.25853 PMC7125023 31702857

[122] Nagashima H Tezuka T Tsuchida W Maeda H Kohroki J Masuho Y. Tandemly repeated fc domain augments binding avidities of antibodies for fcγ receptors, resulting in enhanced antibody-dependent cellular cytotoxicity. Mol Immunol (2008) 45:2752–63. doi: 10.1016/j.molimm.2008.02.003 18353438

[123] Nagashima H Ootsubo M Fukazawa M Motoi S Konakahara S Masuho Y. Enhanced antibody-dependent cellular phagocytosis by chimeric monoclonal antibodies with tandemly repeated fc domains. J Biosci Bioeng (2011) 111:391–6. doi: 10.1016/j.jbiosc.2010.12.007 21215693

[124] Kelton W Mehta N Charab W Lee J Lee C Kojima T. IgGA: A “Cross-isotype” engineered human fc antibody domain that displays both IgG-like and IgA-like effector functions. Chem Biol (2014) 21:1603–9. doi: 10.1016/j.chembiol.2014.10.017 25500223

[125] Jennewein MF Alter G. The immunoregulatory roles of antibody glycosylation. Trends Immunol (2017) 38:358–72. doi: 10.1016/j.it.2017.02.004 28385520

[126] Wu X Guo J Niu M An M Liu L Wang H. Tandem bispecific neutralizing antibody eliminates HIV-1 infection in humanized mice. J Clin Invest (2018) 128:2239–51. doi: 10.1172/JCI96764 PMC5983313 29461979

[127] Khamassi M Xu L Rey J Duchemin M Bouceba T Tuffery P. The CH1α domain of mucosal gp41 IgA contributes to antibody specificity and antiviral functions in HIV-1 highly exposed sero-negative individuals. PLoS Pathog (2020) 16:e1009103. doi: 10.1371/journal.ppat.1009103 33315937 PMC7802955

[128] Marcus R Davies A Ando K Klapper W Opat S Owen C. Obinutuzumab for the first-line treatment of follicular lymphoma. N Engl J Med (2017) 377:1331–44. doi: 10.1056/NEJMoa1614598 28976863

[129] Rochereau N Pavot V Verrier B Ensinas A Genin C Corthésy B. Secretory IgA as a vaccine carrier for delivery of HIV antigen to m cells. Eur J Immunol (2015) 45:773–9. doi: 10.1002/eji.201444816 25412898

[130] Devito C Hinkula J Kaul R Lopalco L Bwayo JJ Plummer F. Mucosal and plasma IgA from HIV-exposed seronegative individuals. Aids (2000) 14:1917–20. 10.1097/00002030-200009080-00006 10997395

[131] Gruell H Klein F. Antibody-mediated prevention and treatment of HIV-1 infection. Retrovirology (2018) 15:73. doi: 10.1186/s12977-018-0455-9 30445968 PMC6240265

[132] Kwon YD Georgiev IS Ofek G Zhang B Asokan M Bailer RT. Optimization of the solubility of HIV-1-Neutralizing antibody 10E8 through somatic variation and structure-based design. J Virol (2016) 90:5899–914. doi: 10.1128/JVI.03246-15 PMC4907239 27053554

[133] Xu L Pegu A Rao E Doria-Rose N Beninga J McKee K. Trispecific broadly neutralizing HIV antibodies mediate potent SHIV protection in macaques. Science (2017) 358:85–90. doi: 10.1126/science.aan8630 28931639 PMC5978417

[134] Wagh K Seaman MS Zingg M Fitzsimons T Barouch DH Burton DR. Potential of conventional & bispecific broadly neutralizing antibodies for prevention of HIV-1 subtype a, c & d infections. PLoS Pathog (2018) 14:e1006860. doi: 10.1371/journal.ppat.1006860 29505593 PMC5854441

[135] Ho DD Huang Y Yu J. Bispecific HIV-1 neutralizing antibodies (2018). Available at: https://patents.google.com/patent/US9884905B2/en (Accessed April 29, 2020).

[136] Rochereau N Pavot V Verrier B Jospin F Ensinas A Genin C. Delivery of antigen to nasal-associated lymphoid tissue microfold cells through secretory IgA targeting local dendritic cells confers protective immunity. J Allergy Clin Immunol (2016) 137:214–222.e2. doi: 10.1016/j.jaci.2015.07.042 26414879

[137] Fouda GG Eudailey J Kunz EL Amos JD Liebl BE Himes J. Systemic administration of an HIV-1 broadly neutralizing dimeric IgA yields mucosal secretory IgA and virus neutralization. Mucosal Immunol (2017) 10:228–37. doi: 10.1038/mi.2016.32 PMC5063654 27072605

[138] Lakhashe SK Amacker M Hariraju D Vyas HK Morrison KS Weiner JA. Cooperation between systemic and mucosal antibodies induced by virosomal vaccines targeting HIV-1 env: Protection of Indian rhesus macaques against low-dose intravaginal SHIV challenges. Front Immunol (2022). doi: 10.3389/fimmu.2022.788619 PMC8902080 35273592

[139] Watkins JD Sholukh AM Mukhtar MM Siddappa NB Lakhashe SK Kim M. Anti-HIV IgA isotypes: Differential virion capture and inhibition of transcytosis are linked to prevention of mucosal R5 SHIV transmission. AIDS Lond Engl (2013) 27:F13–20. doi: 10.1097/QAD.0b013e328360eac6 PMC4084966 23775002

[140] Astronomo RD Santra S Ballweber-Fleming L Westerberg KG Mach L Hensley-McBain T. Neutralization takes precedence over IgG or IgA isotype-related functions in mucosal HIV-1 antibody-mediated protection. EBioMedicine (2016) 14:97–111. doi: 10.1016/j.ebiom.2016.11.024 27919754 PMC5161443

[141] Boesch AW Brown EP Ackerman ME. The role of fc receptors in HIV prevention and therapy. Immunol Rev (2015) 268:296–310. doi: 10.1111/imr.12339 26497529 PMC4955558

[142] Sennepin A Real F Duvivier M Ganor Y Henry S Damotte D. The human penis is a genuine immunological effector site. Front Immunol (2017) 8:1732. doi: 10.3389/fimmu.2017.01732 29312291 PMC5735067

[143] Ehrhardt GRA Davis RS Hsu JT Leu C-M Ehrhardt A Cooper MD. The inhibitory potential of fc receptor homolog 4 on memory b cells. Proc Natl Acad Sci USA (2003) 100:13489–94. doi: 10.1073/pnas.1935944100 PMC263841 14597715

[144] Haga CL Ehrhardt GRA Boohaker RJ Davis RS Cooper MD. Fc receptor-like 5 inhibits b cell activation *via* SHP-1 tyrosine phosphatase recruitment. Proc Natl Acad Sci USA (2007) 104:9770–5. doi: 10.1073/pnas.0703354104 PMC1887609 17522256

[145] Kochi Y Myouzen K Yamada R Suzuki A Kurosaki T Nakamura Y. FCRL3, an autoimmune susceptibility gene, has inhibitory potential on b-cell receptor-mediated signaling. J Immunol Baltim Md 1950 (2009) 183:5502–10. doi: 10.4049/jimmunol.0901982 19843936

[146] Schreeder DM Cannon JP Wu J Li R Shakhmatov MA Davis RS. Cutting edge: FcR-like 6 is an MHC class II receptor. J Immunol Baltim Md 1950 (2010) 185:23–7. doi: 10.4049/jimmunol.1000832 PMC4876727 20519654

[147] Noailly B Yaugel-Novoa M Werquin J Jospin F Drocourt D Bourlet T. Antiviral activities of HIV-1-Specific human broadly neutralizing antibodies are isotype-dependent. Vaccines (Basel) (2022) 10(6):903. doi: 10.3390/vaccines10060903 35746511 PMC9227833

[148] Chu TH Crowley AR Backes I Chang C Tay M Broge T. Hinge length contributes to the phagocytic activity of HIV-specific IgG1 and IgG3 antibodies. PLoS Pathog (2020) 16:e1008083. doi: 10.1371/journal.ppat.1008083 32092122 PMC7058349

